# Geochemical Responses to Natural and Anthropogenic Settings in Salt Lakes Sediments from North-Eastern Romanian Plain

**DOI:** 10.3390/ijerph20020935

**Published:** 2023-01-04

**Authors:** Radu Lucian Olteanu, Cristiana Radulescu, Petre Bretcan, Inga Zinicovscaia, Otilia Culicov, Konstantin Vergel, Danut Tanislav, Marius Bumbac, Cristina Mihaela Nicolescu, Ioana Daniela Dulama, Laura Monica Gorghiu

**Affiliations:** 1 Institute of Multidisciplinary Research for Science and Technology, Valahia University of Targoviste, 130004 Targoviste, Romania; 2 Faculty of Sciences and Arts, Valahia University of Targoviste, 130004 Targoviste, Romania; 3 Doctoral School Chemical Engineering and Biotechnology, Politehnica University of Bucharest, 060042 Bucharest, Romania; 4 Faculty of Humanities, Valahia University of Targoviste, 130105 Targoviste, Romania; 5 Joint Institute for Nuclear Research, 141980 Dubna, Russia; 6 Horia Hulubei National Institute for R&D in Physics and Nuclear Engineering, 077125 Magurele, Romania; 7 National Institute for Research and Development in Electrical Engineering ICPE-CA, 030138 Bucharest, Romania

**Keywords:** salt lake, sediment, NAA, statistical analysis, contamination factor, pollution load index, index of geoaccumulation, enrichment factor

## Abstract

Chemical analysis was performed on sediment core samples collected from three salt lakes, Amara Lake, Caineni Lake, and Movila Miresii Lake, located in the northeast of the Romanian Plain. The concentration of 10 main elements, 6 heavy metals (HMs), 8 rare earth elements (REEs), and 10 trace elements (TEs)—determined using neutron activation analysis (NAA)—showed variability dependent on the depth sections, lake genesis and geochemical characteristics (oxbow, fluvial harbor/liman and loess saucer type). The assessment of pollution indices (contamination factor, pollution load index, geoaccumulation index, and enrichment factor) highlighted low and moderate degrees of contamination for most of the investigated elements. Principal component analysis (PCA) extracted three principal components, explaining 70.33% (Amara Lake), 79.92% (Caineni Lake), and 71.42% (Movila Miresii Lake) of the observed variability. The principal components extracted were assigned to pedological contribution (37.42%—Amara Lake, 55.88%—Caineni Lake, and 15.31%—Movila Miresii Lake), salts depositions (due to the lack of a constant supply of freshwater and through evaporation during dry periods), atmospheric deposition (19.19%—Amara Lake, 13.80%—Caineni Lake, and 10.80%—Movila Miresii Lake), leaching from soil surface/denudation, rock weathering, and mixed anthropogenic input (e.g., agricultural runoff, wastewater discharges) (13.72%—Amara Lake, 10.24%—Caineni Lake, and 45.31%—Movila Miresii Lake).

## 1. Introduction

The main research directions related to lacustrine sediments referred to environmental quality monitoring and paleoenvironmental reconstructions. The transport of polluting substances within the hydrological and ecological systems; the processes that ensure self-purification; the assessment of background pollution, as well as nutrient circuit (N, P), but also the way in which these pollutants affect the balance of aquatic and terrestrial ecosystems are important aspects that are followed carefully in studies of lake sediments [1,2,3,4,5]. As a result of the physicochemical heterogeneity of the sediments, certain sedimentary sections are chosen for analytical investigations in order to determine their chemical composition and the concentration of pollutants [6,7,8,9]. Otherwise, it is known that the distribution of different elements, particularly contaminants, is closely related to the size of the particles, given their affinity for fine particles, a consequence of the adsorption process [10]. Therefore, each sediment sample represents a complex mixture of minerals and organic compounds, to which ions adhere through absorption, adsorption, or complexation reactions [11]. On the contrary, the chemical composition of the sediments represents a function of the characteristics of the hydrographic basin and the lake environment as a whole [7]. The lacustrine (i.e., depositional) environment is under the shaping influence of a complex system of chemical factors, of which the most important are pH, redox potential (Eh), temperature, and salinity [6,12]. These factors—against the background of the influences exerted by the geographical location and the geology of the hydrographic and lacustrine basin, the interaction of the lake with the underground water, the geomorphological processes carried out on the surface of the hydrographic basin, the climate, the vegetation, and human activities—confer the lake sediments distinct chemical properties [13,14]. These can be evaluated by calculating several parameters, such as elemental chemical composition, organic substances (humic compounds, hydrocarbons, amino acids, pigments, etc.), minerals (carbonates, silicates, iron minerals, etc.), acid-volatile sulfides (AVS), and contaminants (heavy metals, pesticides, etc.) [13,15,16]. The elemental chemical composition of lake sediments is characterized by the coexistence of five main groups of chemical elements, with an expected variation from case to case. The first two groups, classified into main elements (Si, Al, K, Na, Mg) and carbonates (Ca, Mg, CO_3_-C), are dominant in most cases because they represent basic structural components in a wide range of minerals from the Earth’s crust [7]. The other groups of chemical elements include nutrients (C_organic_, N, P—10% of the recent sediments matrix), mobile elements (Mn, Fe, S—5% of the total sediment mass), and microelements (Hg, Cd, Pb, Cu, Zn, Ni, Cr, Ag, V, etc.—less than 0.1% of the sediment mass). The concentrations of chemical elements can be maintained as such (represented in the related measurement units on the depth scale in the sedimentary layer) or transformed into accumulation rates and represented on a time scale, depending on the sedimentation rate [17]. Currently, the calculation of different ratios between the concentrations of certain chemical elements in sediments (e.g., Fe:Mn, Zr:Ti, Zr:Rb, etc.) is a widely practiced approach [18,19,20]; however, there were and still are objections related to the multitude of variables that must be taken into account in the interpretation of the obtained results.

The analytical methods used to determine the chemical composition of the sediments and to achieve a complete geochemical stratigraphy have proven their usefulness countless times, providing information related to the source of the sediments; changes in the erosion rate, as well as in the pH and the redox potential; the intensity and impact of atmospheric pollution; the dynamics of nutrients; the impact of deforestation; etc. In addition, due to the fact that the sources of origin of the lake sediment are different, heterogeneous, and difficult to identify, and the material, once deposited in the lake, is subject to diagenetic processes that change its chemical characteristics, the interpretation of the geochemical stratigraphy is difficult to achieve [21,22]. The organic matter in the sediments can be of terrestrial origin (allochthonous), as well as resulting from the natural production of the lake (autochthonous) [4,21]. Neutron activation analysis (NAA), due to its non-destructive nature, multi-elemental character, high accuracy, sensitivity, and reproducibility, is one of the preferred techniques for quantitative analysis of sediments [23].

Sediment contamination with various elements, across a wide range of environments [24,25,26,27], is often assessed using approaches such as contamination factor (CF), pollution load index (PLI), geoaccumulation index (I_geo_), and enrichment factor (EF) [28,29,30,31]. While PLI standardizes the contribution from the elements and is used to indicate bioavailability [30], EF allows identifying natural variations of a given element in order to detect its anthropogenic impact and to find out the extent of the element contaminants to be evaluated in toxicological studies [32,33]. In the estimation of the EF, geochemical elements (e.g., Mn) [24,29,34] are usually used as reference elements to determine anthropogenic contribution.

In this respect, for a good interpretation of geochemical data to characterize the geochemical processes, statistical methods are widely used [3,35,36,37]. Multivariate statistics techniques (e.g., principal component analysis, correlation analysis) are important tools for geochemistry because they reduce a large number of variables to a smaller number of new variables while preserving key features [38,39,40]. These identify elemental associations that are genetically present in a studied mineral deposit/geochemical environment, and they are also applied for the investigation of pollutants in soil, sediment, and natural waters [41,42,43,44]. However, geochemical data are often compositional in nature, which is limited by constant sum representation (namely “closed data”) [45,46,47]. In order to understand the term closed data and, implicitly, better understand the realistic relationships between variables, logarithmic ratio transformations are used [45,47,48,49]. The performed analytical techniques, such as NAA together with multivariate analysis, can lead to a better understanding of the origin and geochemical/geomorphological characteristics of different lakes. It is well known that each lake has a different morphological setting and drainage pattern, with a degree of sedimentation and different geochemical composition, as a consequence of physicochemical processes from sediments [50,51]. These sediments may preserve specific geochemical signatures, which is useful for the interpretation and knowledge of the geomorphological processes of the catchment basin and Earth’s mineral contributions [52,53].

The present study aimed to evaluate the vertical distribution of main elements, potentially toxic heavy metals, rare earth elements, and trace elements and to assess the pollution impact on lakes sediment from Amara Lake, Caineni Lake, and Movila Miresii Lake. The investigated lakes are located in the northeastern part of the Romanian Plain. The objectives of this study were to: (1) investigate the vertical distribution characteristics of 10 main elements, 6 heavy metals (HMs), 8 rare earth elements (REEs) and 10 trace elements (TEs) in salt lakes sediments in southeastern Romania; (2) evaluate the pollution degree of these elements in the investigated area; (3) assess element sources in sediments and potentially drive environmental processes using multivariate statistical methods. The results obtained in this study may provide reliable scientific information for the estimation of the local environmental status and implementation of pollution remediation strategies.

## 2. Materials and Methods

### 2.1. Site Description

**Figure 1:**
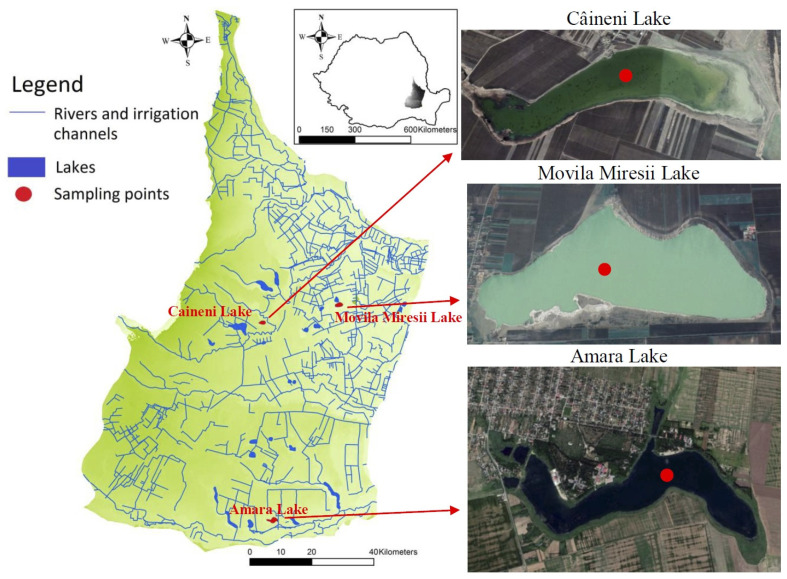
The location of three salt lakes from Romanian Plain and the position related to the sediment cores sampling (data source: left maps—Topographic Map of Romania 1:25,000 actualized and completed; right maps—Google Earth).

The North-Eastern Romanian Plain is part of the Romanian Plain, bounded by the Ialomita, Buzau, Siret, and Danube rivers (Figure 1) and characterized by the large extension of the tabular plains, with low slopes and relief energy. The main hydrographic arteries are represented by allochthonous rivers that also represent the limits of this subunit, to which the Calmatui River is added. The rest of the hydrographic arteries (the autochthonous ones) are small and drain in many cases to endorheic regions, where sinkhole (suffosion depression in loess deposits) lakes can be found. The genesis of the North-Eastern Romanian Plain is closely related to the evolution of the Romanian Plain as a whole, the foundation of the region being largely represented by the Moesian Platform and the Dobrudgean foundation, with a south–north inclination and covered by sediment deposits with thicknesses of 1500–3500 m. Surface Quaternary deposits are represented by clays, sands, gravels, loessoid, and aeolian deposits, next to which there are fluvial deposits with variable thicknesses between 200 and 500 m. Depending on the geographical position, two types of surface deposits are highlighted: some located on lacustrine and aeolian interfluves and those of fluvial origin located in the floodplains of allochthonous rivers.

The climatic conditions are temperate continental, with a manifestation of excess influenced by the circulation of air masses from the east (continental) or south and southwest (Mediterranean origin). Due to the general atmospheric circulation, the relatively flat relief, and homogeneous landscapes, the region is characterized by large contrasts of air temperature between the summer and winter seasons (average annual temperature 10–11°C, maximum temperature recorded in the region of 44.5 °C/10 August 1951, and minimum −32.5 °C/25 January 1942), intense evaporation in summer, and uneven precipitation in time and space (average annual precipitation 450–550 mm). In the present study, the core sediments collected from three salt lakes located in the North-Eastern Romanian Plain were analysed. These lakes were selected because they have small watersheds, are located in endorheic regions, and are genetically different: (1) fluvial liman type; liman is defined in Romanian as an enlarged estuary formed as a lagoon at the wide mouth of one or several rivers, where flow is constrained by a dam of sediments)—Caineni Lake (82 cm sediment core length); (2) loess saucer type—Movila Miresii Lake (90 cm sediment core length) and (3) oxbow type—Amara Lake (94 cm sediment core length). Furthermore, these lakes present anoxic hypolimnetic horizons and density stratification, and the depth at which they are found is correlated with the intake of freshwater, which comes from rainfall, groundwater, or temporary tributaries [4,6,54,55]. The presence and level of different chemical constituents in lake sediments from the Romanian Plain reflect the occurrence and richness of the metals in the rocks of their catchment areas. The change in time of the precipitation regime and of the surface runoff from the hydrographic basin, together with the increase in the thickness of the sediments deposited on the bottom of the lakes, interrupting the connection with the groundwater, leads to changes in the hydrochemical regime. The change in anthropogenic inputs occurred due to the diversification of land use and the changes of natural landscapes through the introduction of agricultural crops [56]. Moreover, the anthropic intervention on the landscapes combined with periods of aridity led to the acceleration of land degradation processes and the increase in the amount of sediment transported by the temporary hydrographic network [14]. The Amara Lake (Slobozia) is located on the terrace of the Ialomita River in a former river branch abandoned and covered with loess [16]; for this reason, some of its features are similar to those of loess saucer lakes. The area of the lake is 1.5 km^2^, with a poorly developed hydrographic basin (45 km^2^), from which it receives two temporary tributaries, the main source of supply being the underground one. The hydrochemical characteristics of the lake are induced by the water sources (surface or underground) and the climatic conditions (temperature, precipitation, evaporation) reflected by the depth regime (maximum depth 3–4 m) (Figure 1). Previous measurements showed a high value of salinity (90 g/L in 1887) when the water level was high and the subsequent reduction of salinity (17 g/L in 1970) due to the increase of water level [55].

The Movila Miresii Lake (Figure 1) is situated in the endorheic region, in which the underground supply water intake is the main component in the hydrological balance and evaporation. Movila Miresii is a loess saucer lake with a mineralization grade, which remarkably varied over the years, such as 138 g/L (1933) to 300 g/L (1960) and then 76.5 g/L (1969) [54,57]. This salt lake (2.52 km^2^ area, 10 m maximum depth, and 11 km^2^ catchment area), with a predominantly underground water supply, accurately reflects the climatic conditions with a strong aridity character of the North-Eastern Romanian Plain.

The Caineni Lake is a fluvial liman (0.1 km^2^ area, maximum depth 1–2 m) with a hydrographic basin of 14.65 km^2^ with intermittent discharge. In 1960, due to the lack of connection with the Buzau River, the salinity of the water was 45.95 g/L; the floods on the Buzau River during 1960–1970 determined the sweetening of the water and the reduction of salinity to 1–2 g/L. After the flood of 1969, Buzau River changed its course and moved 3 km away from Caineni Lake, and a dike was also built. Under these conditions, the salinity registered higher values (9.3 g/L) [16].

### 2.2. Sampling and Sample Preparation

Sediments cores were collected, in 2019, from three different salt lakes (North-Eastern Romanian Plain)—Amara, Movila Miresii, and Caineni—by means of a floating platform and vibrocorer system. The submerged sediments (from shallow depths of water, 1 to 3 m) were extracted using a 1 m long piston with an acrylic core tube. The samples were carried in vertical position in thermally insulated containers at 4 °C and stored in a freezer at −18 °C. The sediment cores were sliced into 2 cm slices/pieces and were dried at 60 °C for 48 h.

### 2.3. Analytical Techniques and Quality Assurance

The elemental profile of analysed sediments was determined using NAA at the REGATA facility of the IBR-2 Reactor (JINR, Dubna, Russia). To determine the content of major, minor, and trace elements, two procedures of samples irradiation were applied. To determine elements with short-lived isotopes (Mg, Al, Ca, Ti, Cl, Mn, and V), after irradiation for 1 min at neutron flux of a thermal neutron flux of 1.6 × 10^12^ n cm^−2^ s^−1^, samples were measured for 15 min. For determination of elements with long-lived isotopes, samples were irradiated for 3 days at a neutron flux of 3.31 × 10^11^ n cm^−2^ and measured twice after 4 (Na, K, Br, As, La, Sm, W and U) and 20 (Fe, Cr, Ni, Co, Zn, Sc, Rb, Sr, Ba, Cs, Sb, Tb, Tm, Yb, Hf, Ta and Th) days for 30 and 90 min, respectively. Gamma spectra were recorded using HPGe detectors, with a 1.9 keV resolution for the ^60^Co 1332 keV line, and processed using Genie 2000 software. Elements content in samples was calculated by applying software developed in JINR. A set of certified reference materials provided by the National Institute of Standards and Technology (USA)—NIST 2710 Montana Soil, NIST 1633c Coal fly ash, NIST 2709 Trace elements in soil, NIST 1633b Coal fly ash, NIST 1632c Trace elements in coal, NIST 1575A Pine needles, NIST 1547 Peach leaves, and BCR 667 Estuarine sediment—were irradiated in the same conditions with samples to ensure the quality of the measurements. The difference between determined and certified values was in the range 3–7%, which is in accordance with previously performed studies [58,59]. The validation of NAA data is proved by inter-laboratory studies, such as the Worldwide Open Proficiency Test for Nuclear and Related Analytical Techniques Laboratories for soil samples [60].

### 2.4. Statistical Analysis

#### 2.4.1. Assessment of Sediment Core Contamination

The assessment of the sediment core pollution by heavy metals (HM), trace elements (TE), and rare earth elements (REE) was made on the basis of four commonly used indices: the contamination factor (CF), the pollution load index (PLI), the index of geoaccumulation (I_geo_), and the enrichment factor (EF). The equation and the evaluation criteria for assessing the pollution indices are presented in Table 1. Manganese (Mn) was used as a reference element in the estimation of EF [24,29,34] in order to evaluate the anthropogenic element enrichment. CF, I_geo_, and EF are single indices, while PLI is an integrated index. For I_geo_, EF, and CF, the concentrations of HM, TEs, and REEs in sediments are related to the geochemical background element value (GBV). Because GBVs for the sediments from the study area are not available, the calculated values were used in the present work to assess the pollution indices. The limit values for the assessment of sediment contamination have been assumed from freshwater sediment quality guidelines (SQGs) [61,62].

#### 2.4.2. Multivariate Statistical Approach

Pearson correlation analysis and principal component analysis (PCA) were used to identify the possible sources of elements in sediment samples and to reduce a large number of variables to a smaller number of new variables while preserving key features. Varimax normalized PCA (Kaiser Normalization) was used to determine the association of the different variables (the concentrations of the quantified elements from sediment samples) by reducing the dimensionality of the dataset. The validity of the PCA was checked with the Kaiser–Meyer–Olkin measure of sampling adequacy (KMO) and Bartlett’s test. The PCA was performed to determine possible chemical associations that could explain element behaviors and the relationships between them. The first three principal components (PCs) or factors (F) with eigenvalues greater than one were considered to be relevant and were further used in the analysis [66].

In order to combine the geochemical information retrieved from sediment sections, an agglomerative hierarchical clustering (AHC) analysis was performed for sediment core sections, in which elemental concentration was determined. AHC is an iterative classification method that initiates clustering by calculating the dissimilarity between different groups of objects; cluster analysis algorithms made it possible to cluster the objects together to minimize a given agglomeration criterion [38,67,68,69,70]. The resulting dendrogram representation was easier to interpret than the intricate variability of the raw dataset. The Hartigan index used, in order not to have to define an arbitrary number of clusters, allows comparing the quality of several clusters according to a defined number of clusters (between 2 and 5); the Hartigan index will automatically define the appropriate number of clusters within this range. AHC was carried out by using Ward’s method, with the Euclidean distance (proximity matrix) as a measure of dissimilarity to assess the associations among the sediment sections (vertical distribution) with respect to the element concentrations. Data processing was done using XLSTAT 2022.4.1 (Addinsoft 2022) statistical and data analysis software [71].

## 3. Results and Discussion

### 3.1. Element Concentrations in the Amara Lake Sediment Core

The results of the analysis of 34 elements show for most of them a distinct pattern, in which some of them occur in the largest amount in the upper part of the sediment core, down to 20 cm, and occur in much lower concentrations below this depth. Other elements show an opposite trend, occurring in much smaller amounts in the upper 20 cm, and have their highest concentration under this level. In addition, some elements do not fit any of these two main patterns mentioned above; other trends can also be distinguished within these two main patterns. The section/border between 17 and 21 cm seems to be significant for understanding the chemical stratigraphy of the investigated sediment core.

**Figure 2:**
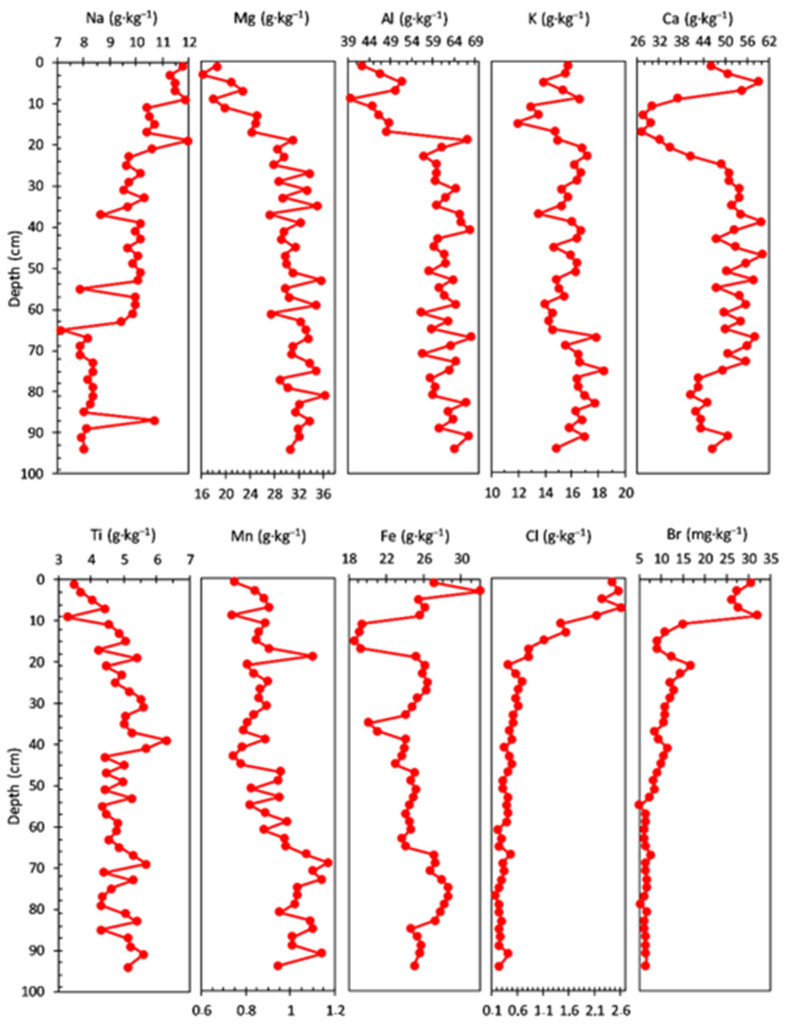
Vertical distribution of the main geochemical elements (Na, Mg, Al, K, Ca, Ti, Mn, Fe, Cl, Br) in the Amara Lake sediment core (94 cm depth).

**Figure 3:**
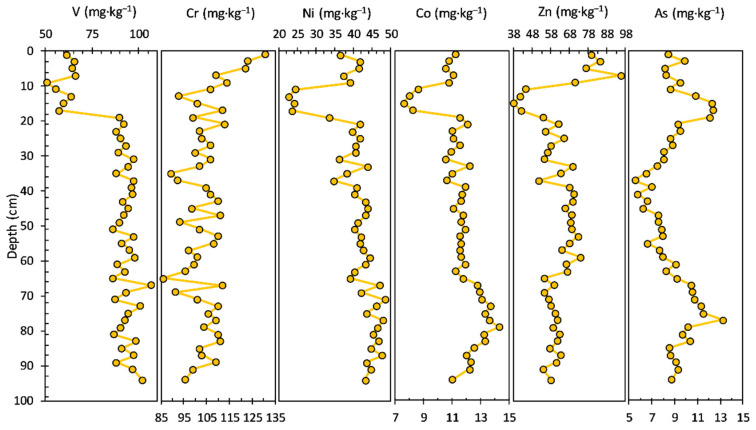
Vertical distribution of geochemical heavy metals (V, Cr, Ni, Co, Zn, As) in the Amara Lake sediment core (94 cm depth).

**Figure 4:**
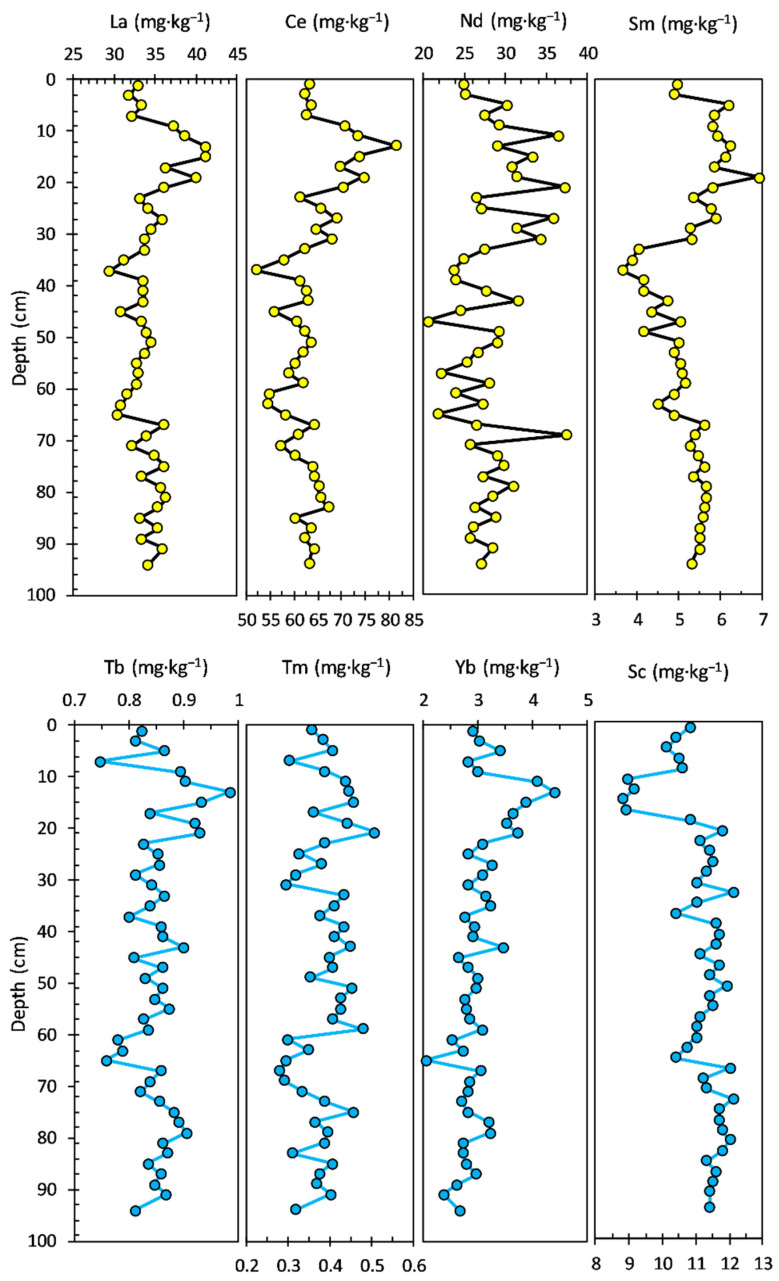
Vertical distribution of geochemical light rare earth elements—LREE (La, Ce, Nd, Sm) and heavy rare earth elements—HREE (Tb, Tm, Yb, Sc) in the Amara Lake sediment core (94 cm depth).

**Figure 5:**
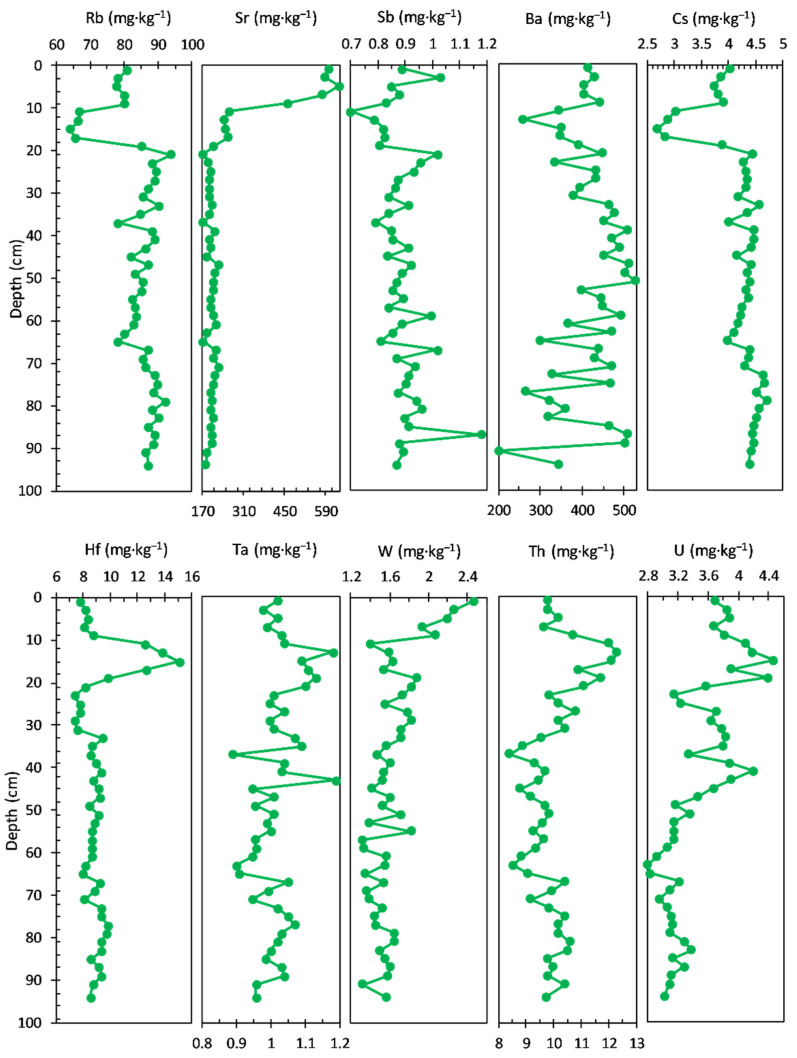
Vertical distribution of geochemical trace elements (Rb, Sr, Sb, Ba, Cs, Hf, Ta, W, Th, U) in the Lake Amara sediment core (94 cm depth).

The concentration profiles are presented in Figure 2, Figure 3, Figure 4 and Figure 5, with the analysed elements divided into main elements, heavy metals (HM), light rare earth elements (LREE), heavy rare earth elements (HREE), and trace elements (TE).

The concentrations of main elements measured in the sediment core are shown in Figure 2. The Na concentration ranged from 7.17 to 12.00 g·kg^−1^, with an average value of 9.60 g·kg^−1^; a gradual increase in concentration with three levels of variation structured on the depth, from the bottom to the surface, of the sediment core can be noticed: 94–65 cm (average value of 8.25 g·kg^−1^), 63–23 cm (average value of 9.77 g·kg^−1^), and 21–1.0 cm (average value of 11.14 g·kg^−1^). The concentration of Mg had a range from 16.20 to 36.30 g·kg^−1^, with an average value of 29.40 g·kg^−1^; a variation of the concentration can be observed on two depth levels, from the bottom to the surface, of the sediment core, with a gradual decrease in the second (surface) level: 94–19 cm (average value of 31.36 g·kg^−1^) and 17–1.0 cm (average value of 21.12 g·kg^−1^).

The Al concentration profile (39.50 to 68.10 g·kg^−1^ range, with an average value of 59.04 g·kg^−1^) has a similar trend with that of Mg observed on two depth levels, from the bottom to the surface, of the sediment core: 94–19 cm (average value of 62.02 g·kg^−1^) and 17–1.0 cm (average value of 46.44 g·kg^−1^).

The concentration of K had a range from 11.90 to 18.40 g·kg^−1^, with an average value of 15.60 g·kg^−1^, and a relatively similar trend with that of Na (three levels of variation structured on depth, from the bottom to the surface) in the sediment core: 94–67 cm (average value of 16.59 g·kg^−1^), 65–17 cm (average value of 15.44 g·kg^−1^), and 15–1.0 cm (average value of 14.38 g·kg^−1^).

The Ca concentration profile ranged from 27.20 to 60.20 g·kg^−1^, with an average value of 47.97 g·kg^−1^; four levels of variation (from the bottom to the surface of the sediment core) can be noticed: 94–77 cm (average value of 44.24 g·kg^−1^), 75–25 cm (average value of 53.30 g·kg^−1^), 23–11 cm (average value of 31.77 g·kg^−1^), and 9–1.0 cm (average value of 49.68 g·kg^−1^).

A similar variation (a notable decrease in the sediment core) with Ca concentration in the 23–11 cm layer is also observed for Al, Ni, Co, Zn, and Fe; this pattern suggests a significant reduction in pedological input. The decrease in the content of Fe correlated with that of Ca may be due to the intensification of the reducing conditions in the hypolimnion (which increases the solubility of Fe), due either to the accumulation of humus in a large amount or to the existence of a stratification of water. The content of Sr does not register, in the same sediment layer, a significant variation compared to Ca; as a result, the increase in the Sr:Ca ratio indicates a progressive increase in salinity, also observed by the variation in the content of Cl and Br (it must be taken into account that part of the Sr can come from the aeolian input of clastic material).

The concentration of Ti ranged from 3.28 to 6.29 g·kg^−1^, with an average value of 4.81 g·kg^−1^; a variation of the concentration can be observed on two depth levels, from the bottom to the surface, of the sediment core, with a gradual decrease in the second (surface) level: 94–11 cm (average value of 4.94 g·kg^−1^) and 9.0–1.0 cm (average value of 3.78 g·kg^−1^).

Both the Mn and Fe profile concentrations share almost a similar trend, except younger sediment layers (17–1.0 cm depth), where Fe (average value 23.62 g·kg^−1^) and Mn (average value 0.84 g·kg^−1^) concentrations increase and decrease (from the bottom to the surface), respectively; a relatively small variation of the concentration can be observed also for both elements on three depth levels, from the bottom to the surface, of the sediment core: 94–85 cm (average values of 1.04 g·kg^−1^ for Mn and 25.22 g·kg^−1^ for Fe), 83–67 cm (average values of 1.06 g·kg^−1^ for Mn and 27.67 g·kg^−1^ for Fe), and 65–19 cm (average values of 0.87 g·kg^−1^ for Mn and 24.36 g·kg^−1^ for Fe). Surface processes, diagenetic transformation, and humic matter interactions influence the net burial rates of Fe and Mn and can obscure the interpretation of Mn/Fe ratio as a redox proxy [72]. Residual Fe and Mn, and crystalline Fe oxy(hydr)oxides and Mn oxides, are primarily sourced from the catchment soils, whereas humic Fe and Mn is related to dissolved organic carbon in the lake and leaching from soils and wetlands. Fe and Mn seem to behave similarly to catchment processes, though Fe accumulation is considerably higher; crystalline Fe oxy(hydr)oxides and Mn oxides are generally stable under all prevailing redox conditions [72,73]. The Mn/Fe ratio is lower, mainly due to the higher allogenic input of the crystalline Fe oxy(hydr)oxides in the sediments. Periods of higher detrital input effects can be distinguished and corrected by normalization with Ti [74,75], as can be seen in the Fe/Ti and Mn/Ti ratios [72].

The concentration profile of Cl (0.17 to 2.63 g·kg^−1^ range, with an average value of 0.68 g·kg^−1^) and Br (0.17 to 2.63 mg·kg^−1^ range, with an average value of 0.68 mg·kg^−1^) have almost a similar pattern, with three depth levels, from the bottom to the surface, of the sediment core: 94–55 cm (average values of 0.30 g·kg^−1^ for Cl and 6.45 mg·kg^−1^ for Br), 53–21 cm (average values of 0.48 g·kg^−1^ for Cl and 10.92 mg·kg^−1^ for Br), and 19–1.0 cm (average values of 1.78 g·kg^−1^ for Cl and 20.06 mg·kg^−1^ for Br). For both Cl and Br, a significant increase of concentration can be observed in the young sediment layers (the last level from the surface previously mentioned).

The concentrations of heavy metals/potentially toxic elements measured in the sediment core are shown in Figure 3. The V concentration ranged from 51.20 to 107.00 mg·kg^−1^, with an average value of 87.08 mg·kg^−1^; a decrease in concentration in the upper layers of the sediment core can be noticed: 94–19 cm (average value of 93.91 mg·kg^−1^) and 17–1.0 cm (average value of 60.79 mg·kg^−1^).

The concentration of Cr had a range from 86.10 to 131.00 mg·kg^−1^, with an average value of 104.58 mg·kg^−1^; an increase in concentration can be seen in the upper layers of the sediment core: 94–17 cm (average value of 102.91 mg·kg^−1^) and 15–1.0 cm (average value of 114.11 mg·kg^−1^).

The concentration profiles of Ni (22.60 to 48.30 mg·kg^−1^ concentration range, with an average value of 40.45 mg·kg^−1^) and Co (7.58 to 14.30 mg·kg^−1^ concentration range, with an average value of 11.53 mg·kg^−1^) share a similar pattern from the bottom to the surface of the sediment core: 94–21 cm (Ni—average value of 42.63 mg·kg^−1^ and Co—average value of 11.98 mg·kg^−1^), 17–11 cm (Ni—average value of 23.68 mg·kg^−1^ and Co—average value of 8.09 mg·kg^−1^), and 9–1.0 cm (Ni—average value of 39.14 mg·kg^−1^ and Co—average value of 10.88 mg·kg^−1^). Similarities in the sediment core variability of iron and manganese (especially iron) and heavy metals like Ni, Co, and Zn indicate sorption or co-precipitation of these elements with Fe and Mn hydroxides and oxides, as well as sorption by clay minerals, chemical processes involving hydrolytic reactions and both complexed and dissolved ions. Zn, V, Ni, and Co can be linked mostly to the lake catchment rocks, implying a source-rock effect controlling the vertical distribution of these elements, especially in the lower bottom layers.

The Zn concentration ranged from 38.00 to 95.40 mg·kg^−1^, with an average value of 62.42 mg·kg^−1^; the concentration profile looks almost similar to those of Ni and Co. The concentration of As had a range from 5.53 to 13.20 mg·kg^−1^, with an average value of 8.62 mg·kg^−1^. Most of the potentially toxic elements, including V, Ni, Co, and Zn, exhibited similar distribution patterns to Al in the bottom sediment layers (94–19 cm). The concentration profiles of LREEs and HREEs in the sediment core are shown in Figure 4. Most of the LREEs, including La, Ce, and Sm, exhibited similar distribution patterns. The La concentration ranged from 29.60 to 41.20 mg·kg^−1^, with an average value of 34.33 mg·kg^−1^. The concentration of Ce had a range from 52.00 to 81.40 mg·kg^−1^, with an average value of 63.44 mg·kg^−1^. Nd and Sm concentrations ranged from 20.50 to 37.40 mg·kg^−1^, with an average value of 28.17 mg·kg^−1^ and from 3.64 to 6.89 mg·kg^−1^, with an average value of 5.23 mg·kg^−1^, respectively. HREEs show a similar pattern of concentration profiles in the bottom section (94–23 cm) of the sediment core, and visible differences can be observed in the younger layers of the sediment core.

The Tb concentration ranged from 0.75 to 0.98 mg·kg^−1^, with an average value of 0.85 mg·kg^−1^. The concentration profiles of Tm (0.28 to 0.50 mg·kg^−1^ range, with an average value of 0.39 mg·kg^−1^) and Yb (2.05 to 4.39 mg·kg^−1^ range, with an average value of 3.01 mg·kg^−1^) looks almost similar to that of Tb, even in upper layers of the sediment core. The concentration of Sc had a range from 8.81 to 12.10 mg·kg^−1^, with an average value of 11.10 mg·kg^−1^.

The concentrations of TEs measured in the sediment core are shown in Figure 5. Rb (64.30 to 93.80 mg·kg^−1^ range, with an average value of 84.13 mg·kg^−1^) and Cs (2.69 to 4.70 mg·kg^−1^ range, with an average value of 4.17 mg·kg^−1^) concentrations share a similar pattern, with almost stable values in the bottom sediment core (94–19 cm) and variations in the upper layers (17–1.0 cm). From trace elements, Sr (174.00 to 638.00 mg·kg^−1^ range, with an average value of 245.38 mg·kg^−1^) and Hf (7.38 to 15.10 mg·kg^−1^ range, with an average value of 9.11 mg·kg^−1^) show also a similar concentration profile in the bottom sediment cores (Sr—average value of 201.08 mg·kg^−1^ and Hf—average value of 8.76 mg·kg^−1^). Significant variations can be seen in the upper layers: 17–11 cm (Sr—average value of 253.00 mg·kg^−1^ and Hf—average value of 13.55 mg·kg^−1^, with a maximum value at about 16 cm) and 9–1.0 cm (Sr—average value of 574.00 mg·kg^−1^, reaching a maximum at about 6 cm and Hf—average value of 8.22 mg·kg^−1^).

The concentration profiles of Sb, Ba, Ta, and W do not present distinct features over the sediment core, except for the increase of W concentration in the upper layer: Sb—0.70 to 1.18 mg·kg^−1^ range, with an average value of 0.89 mg·kg^−1^; Ba—201.00 to 528.00 mg·kg^−1^ range, with an average value of 412.79 mg·kg^−1^; Ta—0.89 to 1.19 mg·kg^−1^ range, with an average value of 1.02 mg·kg^−1^, and W—1.32 to 2.46 mg·kg^−1^ range, with an average value of 1.63 mg·kg^−1^. Two elements—Th (8.42 to 12.30 mg·kg^−1^ range, with an average value of 10.00 mg·kg^−1^) and U (2.80 to 4.47 mg·kg^−1^ range, with an average value of 3.46 mg·kg^−1^)—exhibit almost similar trends over the entire core sediment.

### 3.2. Element Concentrations in the Caineni Lake Sediment Core

**Figure 6:**
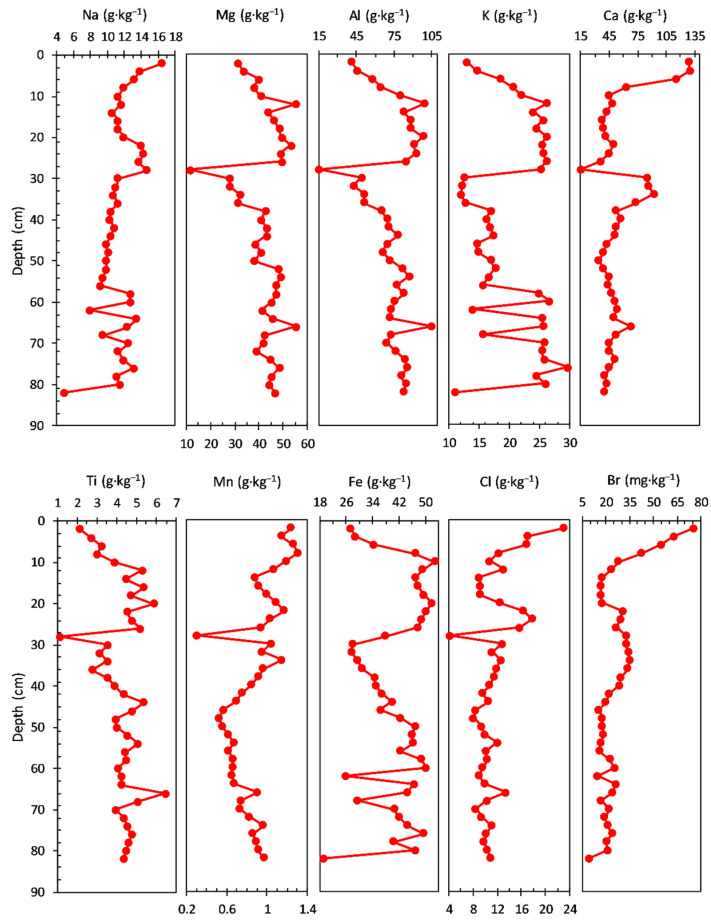
Vertical distribution of geochemical main elements (Na, Mg, Al, K, Ca, Ti, Mn, Fe, Cl, Br) in the Lake Caineni sediment core (82 cm depth).

**Figure 7:**
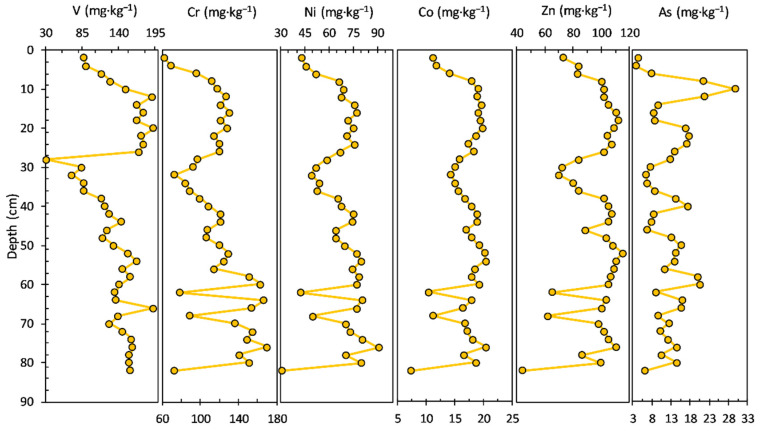
Vertical distribution of geochemical heavy metals (V, Cr, Ni, Co, Zn, As) in the Caineni Lake sediment core (82 cm depth).

**Figure 8:**
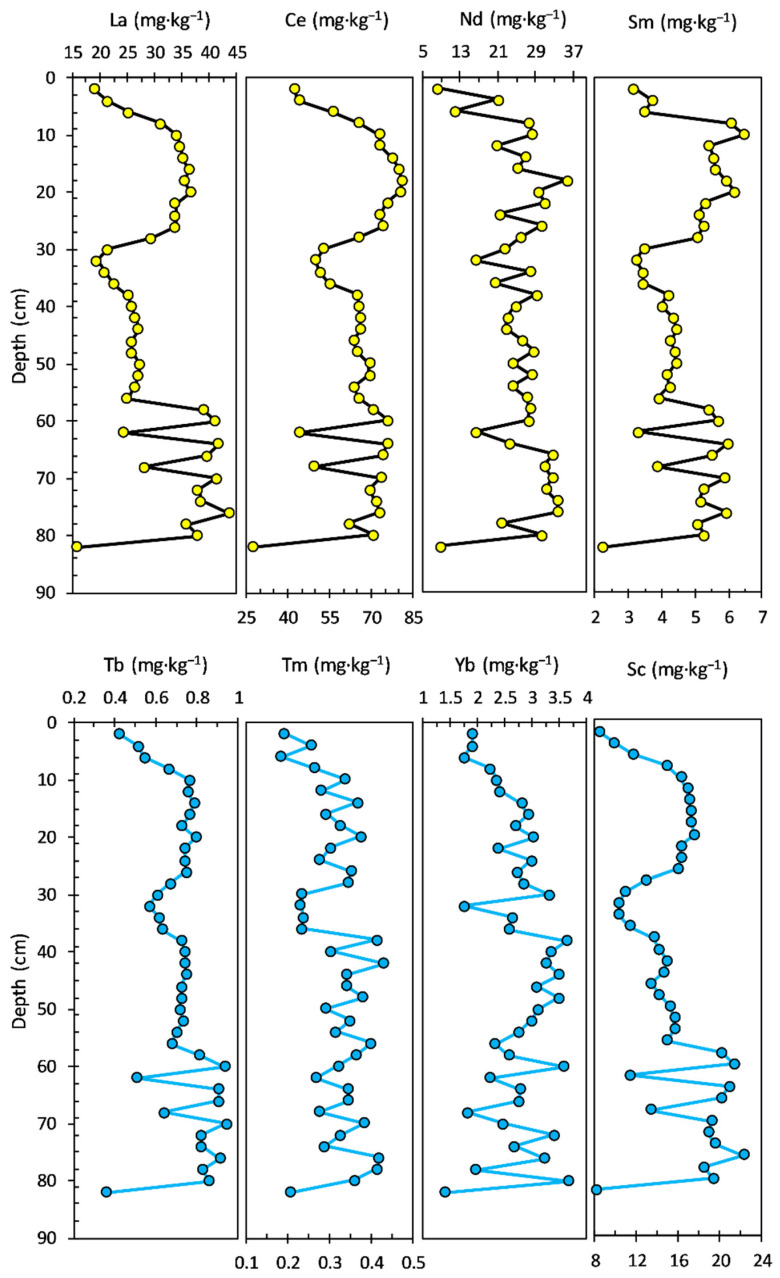
Vertical distribution of geochemical light rare earth elements—LREE (La, Ce, Nd, Sm) and heavy rare earth elements—HREE (Tb, Tm, Yb, Sc) in the Caineni Lake sediment core (82 cm depth).

**Figure 9:**
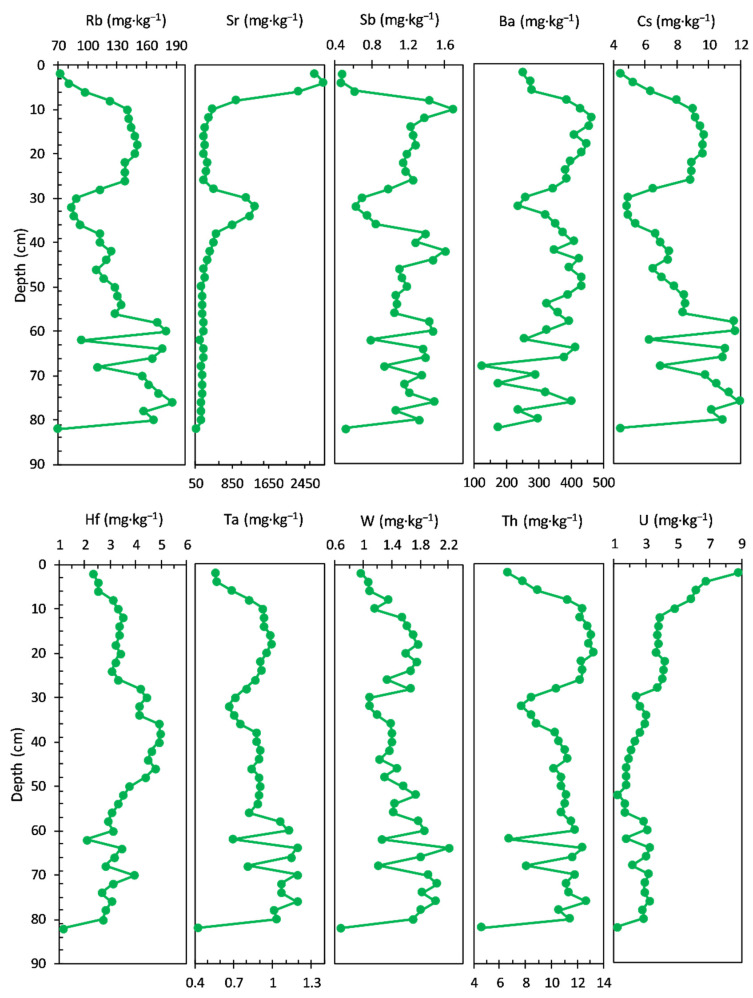
Vertical distribution of geochemical trace elements (Rb, Sr, Sb, Ba, Cs, Hf, Ta, W, Th, U) in the Caineni Lake sediment core (82 cm depth).

The results of the analysis of the main elements HM, LREE, and HREE and the trace elements show for most of them a distinct pattern, in which some of them occur in the smallest amount in the upper part of the sediment core, down to 10–12 cm, and occur in much higher concentrations below this depth. Other elements show an opposite trend, occurring in much higher amounts in the upper layer, and they have their highest concentration under this level. Additionally, some elements do not fit any of these two main patterns mentioned above; other trends can also be distinguished within these two main patterns. The borders between 28 and 32 cm and 58 and 54 cm seem to be significant to understanding the chemical stratigraphy of the investigated sediment core. The concentration profiles for the main elements, heavy metals (HM), light rare earth elements (LREE), heavy rare earth elements (HREE), and trace elements (TE) are presented in Figure 6, Figure 7, Figure 8 and Figure 9.

The concentrations of the main elements measured in the sediment core are shown in Figure 6. The Na concentration ranged from 4.97 to 16.40 g·kg^−1^, with an average value of 11.45 g·kg^−1^; a gradual increase in concentration structured on the depth, from the bottom to the surface, of the sediment core can be noticed: 82–58 cm (average value of 11.16 g·kg^−1^; notable variations between analysed sections), 56–30 cm (average value of 9.77 g·kg^−1^; uniform variation with gradual increase), 28–20 cm (average value of 13.74 g·kg^−1^; uniform variation with gradual decrease), 18–10 cm (average value of 11.20 g·kg^−1^; constant variation trend), and 8–0 cm (average value of 13.80 g·kg^−1^; gradual increase in uppermost sediment layer).

The Mg (ranged from 11.20 to 55.20 g·kg^−1^, with an average value of 42.03 g·kg^−1^) and Al (ranged from 15.20 to 105.00 g·kg^−1^, with an average value of 73.21 g·kg^−1^) concentration profiles display a similar trend structured on three levels: 82–38 cm (Mg—average value of 44.19 g·kg^−1^ and Al—average value of 77.33 g·kg^−1^, with almost constant variation between sections), 36–28 cm (Mg—average value of 26.06 g·kg^−1^ and Al—average value of 41.82 g·kg^−1^, with gradual decrease and a minimum value at 28 cm for both elements), and 26–0.0 cm (gradual decrease of concentration; Mg—average value of 44.37 g·kg^−1^ and Al—average value of 78.01 g·kg^−1^).

The notable Mg and Al variation at 28 cm depth in the sediment core suggests a possible pH-dependent dissolution of aluminium hydroxides to form aluminate (process present in shallow lakes, such as the present one) where resuspension at pH increase in the water occurs frequently. The decrease in Fe and especially Mn content, at the same time, may be due to the presence of reducing conditions (which increase the solubility of Fe and Mn; manganese is more soluble than iron). Furthermore, the same sudden variation observed for Ca, Ti, Cl, and V suggests a significant supply of fresh water.

In addition, the K geochemical profile (ranged from 10.90 to 29.60 g·kg^−1^, with an average value of 20.21 g·kg^−1^) shows a similar variation to that of Na in the bottom sediment layers (82–58 cm) and an opposite trend in the upper layers (56–0 cm): 82–58 cm (average value of 23.02 g·kg^−1^; notable variations between analysed sections), 56–30 cm (average value of 15.19 g·kg^−1^; uniform variation with gradual decrease), 28–12 cm (average value of 15.19 g·kg^−1^; constant variation), and 10–0 cm (average value of 17.68 g·kg^−1^; significant decrease in the youngest sediment sections).

The concentration profile of Ca (ranged from 15.50 to 130.00 g·kg^−1^, with an average value of 55.38 g·kg^−1^) displays almost a constant variation in the bottom sediment layers, with notable changes in the upper layers (38–0 cm): 82–38 cm (average value of 47.43 g·kg^−1^; gradual slow increase), 36–30 cm (average value of 84.58 g·kg^−1^; constant variation), 28–8 cm (average value of 42.40 g·kg^−1^; gradual increase in variation), and 6.0–0.0 cm (average value of 125.00 g·kg^−1^).

The Ti concentration (ranged from 1.15 to 6.44 g·kg^−1^, with an average value of 4.21 g·kg^−1^) presents almost a similar pattern as Mg and Al: 82–30 cm (average value of 4.31 g·kg^−1^, with maximum and minimum values at 66 cm and 28 cm, respectively), 26–0.0 cm (average value of 4.24 g·kg^−1^; gradual decrease).

The concentration profile of Mn (ranged from 0.30 to 1.31 g·kg^−1^, with an average value of 0.87 g·kg^−1^) follows almost the same pattern (except for the 44–36 cm section core) in the bottom sediment layers with Mg, Al, and Ti: 82–48 cm (average value of 0.75 g·kg^−1^; gradual decrease), 46–22 cm (average value of 0.87 g·kg^−1^; gradual increase with a minimum at 28 cm/0.30 g·kg^−1^), 20–14 cm (average value of 0.97 g·kg^−1^), and 12–0 cm (average value of 1.20 g·kg^−1^).

The Fe concentration (ranged from 19.3 to 52.9 g·kg^−1^, with an average value of 40.76 g·kg^−1^) displays a similar trend as K on the depth of the sediment core: 82–54 cm (average value of 41.15 g·kg^−1^), 52–32 cm (average value of 36.29 g·kg^−1^; uniform variation with gradual decrease), 26–10 cm (average value of 49.41 g·kg^−1^; almost constant variation), and 8–0 cm (average value of 30.17 g·kg^−1^; gradual decrease).

Both concentration profiles of Cl (ranged from 4.16 to 23.00 g·kg^−1^, with an average value of 11.43 g·kg^−1^) and Br (ranged from 9.27 to 75.70 mg·kg^−1^, with an average value of 26.43 mg·kg^−1^) show a similar trend of variation. On the contrary, the Cl concentration displays notable variations, especially in the upper layers of the sediment core: 82–30 cm (average value of 4.31 g·kg^−1^; almost constant between analysed sections), 24–18 cm (average value of 13.91 g·kg^−1^; gradual decrease), and 16–0 cm (average value of 13.91 g·kg^−1^; gradual increase). Moreover, for the Br concentration profile, notable variations can be observed in the upper layers of the sediment core: 82–14 cm (average value of 22.77 mg·kg^−1^; almost constant between analysed sections) and 12–0.0 cm (average value of 47.83 mg·kg^−1^; gradual increase).

The concentration profiles of the heavy metals (V, Cr, Ni, Co, Zn, and As) measured in the sediment core are shown in Figure 7. In this respect, it can be seen from Figure 7 that V and particularly As display a different pattern relative to the rest of the analysed HM (Cr, Ni, Co, and Zn show almost a similar trend on the depth of the sediment core). In addition, the V concentration profile (ranged from 31.30 to 193.00 mg·kg^−1^, with an average value of 138.35 mg·kg^−1^), from the bottom to the surface of the sediment core, shows a relative slow variation (decrease), followed by a notable increase in the upper layers: 82–30 cm (average value of 135.09 mg·kg^−1^; slow gradual decrease), 26–12 cm (average value of 177.50 mg·kg^−1^; constant variation), and 10–0.0 cm (average value of 114.74 mg·kg^−1^; uniform variation with gradual decrease).

The concentration profile of As (ranged from 3.72 to 29.60 mg·kg^−1^, with an average value of 12.56 mg·kg^−1^) shows a similar variation as the rest of the HM, mostly in the 46–14 cm sediment core, with significant variations noticed almost all along the analysed sections: 82–14 cm (average value of 12.18 mg·kg^−1^) and 10–0.0 cm (average value of 13.37 mg·kg^−1^). Cr (ranged from 62.30 to 170.00 mg·kg^−1^, with an average value of 117.39 mg·kg^−1^), Ni (ranged from 3.05 to 90.08 mg·kg^−1^, with an average value of 66.74 mg·kg^−1^), Co (ranged from 7.39 to 20.04 mg·kg^−1^, with an average value of 16.91 mg·kg^−1^), and Zn (ranged from 44.1 to 115.00 mg·kg^−1^, with an average value of 95.59 mg·kg^−1^) concentrations present a similar pattern in the following core sections: 82–56 cm (Cr—average value of 135.16 mg·kg^−1^; Ni—average value of 67.90 mg·kg^−1^; Co—average value of 16.14 mg·kg^−1^; Zn—average value of 92.44 mg·kg^−1^), 54–32 cm (Cr—average value of 117.28 mg·kg^−1^; Ni—average value of 66.11 mg·kg^−1^; Co—average value of 17.65 mg·kg^−1^; Zn—average value of 98.06 mg·kg^−1^), 26–12 cm (Cr—average value of 123.13 mg·kg^−1^; Ni—average value of 72.65 mg·kg^−1^; Co—average value of 18.25 mg·kg^−1^; Zn—average value of 106.38 mg·kg^−1^), and 10–0.0 cm (Cr—average value of 91.60 mg·kg^−1^; Ni—average value of 55.16 mg·kg^−1^; Co—average value of 14.78 mg·kg^−1^; Zn—average value of 88.28 mg·kg^−1^).

The vertical distribution of LREEs and HREEs concentrations in the sediment core is illustrated in Figure 8. All four LREEs present almost a similar concentration profile, with the main differences noticed for Nd and Sm in the upper sediment layers. La (ranged from 15.90 to 43.70 mg·kg^−1^, with an average value of 30.53 mg·kg^−1^) and Ce (ranged from 27.10 to 80.90 mg·kg^−1^, with an average value of 64.95 mg·kg^−1^) concentration profiles show four notable sections, starting from the bottom sediment core: 82–58 cm (La—average value of 35.78 mg·kg^−1^ and Ce—average value of 64.32 mg·kg^−1^; significant variation between sections), 56–30 cm (La—average value of 24.74 mg·kg^−1^ and Ce—average value of 61.91 mg·kg^−1^), 28–8 cm (La—average value of 34.04 mg·kg^−1^ and Ce—average value of 74.35 mg·kg^−1^), and 6–0.0 cm (La—average value of 21.93 mg·kg^−1^ and Ce—average value of 47.33 mg·kg^−1^).

The concentration of Nd (ranged from 8.22 to 35.60 mg·kg^−1^, with an average value of 25.34 mg·kg^−1^) displays a notable variation, especially in the upper and bottom sediment core layers: 82–58 cm (average value of 29.26 mg·kg^−1^) and 56–0 cm (average value of 24.59 mg·kg^−1^). The Sm profile (ranged from 2.21 to 6.47 mg·kg^−1^, with an average value of 4.69 mg·kg^−1^) shows small differences relative to La and Ce, mostly in the upper layers of the sediment core: 28–8 cm (average value of 5.62 mg·kg^−1^) and 6–0 cm (average value of 3.44 mg·kg^−1^).

The concentration profile of HREEs follows generally the same pattern (except for Tm and Yb) as LREEs. Tb (ranged from 0.36 to 0.94 mg·kg^−1^, with an average value of 0.72 mg·kg^−1^) and Sc (ranged from 8.06 to 22.20 mg·kg^−1^, with an average value of 15.34 mg·kg^−1^) concentrations display almost the same profile along the sediment core: 82–58 cm (Tb—average value of 0.79 mg·kg^−1^ and Sc—average value of 17.87 mg·kg^−1^; significant variation between sections), 56–10 cm (Tb—average value of 0.71 mg·kg^−1^ and Sc—average value of 14.66 mg·kg^−1^; small variations between sections), and 8–0 cm (Tb—average value of 0.54 mg·kg^−1^ and Sc—average value of 11.16 mg·kg^−1^).

The concentration profile of Tm (ranged from 0.19 to 0.43 mg·kg^−1^, with an average value of 0.32 mg·kg^−1^) shows a nonspecific variation, with a constant trend noticed only in the 36–30 cm range (average value of 0.23 mg·kg^−1^). The concentration of Yb (ranged from 1.41 to 3.67 mg·kg^−1^, with an average value of 2.71 mg·kg^−1^) also displays nonspecific variation, with a general profile close to that of Tm.

The concentration profiles of the trace elements measured in the sediment core are shown in Figure 9. Sr and U display a different pattern relative to the rest of analysed elements. On the contrary, the other elements display generally the same trend, with most similarities noticed between Rb (ranged from 70.50 to 187.00 mg·kg^−1^, with an average value of 129.97 mg·kg^−1^), Cs (ranged from 4.38 to 11.90 mg·kg^−1^, with an average value of 8.15 mg·kg^−1^), Ba (ranged from 123.00 to 464.00 mg·kg^−1^, with an average value of 345.61 mg·kg^−1^), and Sb (ranged from 0.47 to 1.70 mg·kg^−1^, with an average value of 1.13 mg·kg^−1^).

However, the concentration profiles of Hf (ranged from 1.17 to 4.93 mg·kg^−1^, with an average value of 3.42 mg·kg^−1^), Ta (ranged from 0.42 to 1.19 mg·kg^−1^, with an average value of 0.89 mg·kg^−1^), Th (ranged from 4.65 to 13.30 mg·kg^−1^, with an average value of 10.62 mg·kg^−1^), and W (ranged from 0.68 to 2.21 mg·kg^−1^, with an average value of 1.50 mg·kg^−1^) allow them to be relatively differentiated from the previous group.

In addition, the Sr concentration (ranged from 74.30 to 2870.00 mg·kg^−1^, with an average value of 534.20 mg·kg^−1^) profile, from the bottom to the surface of the sediment core, shows a relatively slow variation, followed by a notable increase in the upper layers: 82–38 cm (average value of 235.71 mg·kg^−1^), 36–28 cm (maximum at 32 cm/1360.00 mg·kg^−1^), 28–10 cm (average value of 306.00 mg·kg^−1^), and 8–0 cm (average value of 2201.75 mg·kg^−1^; significant increase in most upper layer).

The concentration profile of U (ranged from 1.19 to 8.77 mg·kg^−1^, with an average value of 3.21 mg·kg^−1^) shows gradual increase from the bottom to the upper layers of the sediment core: 82–12 cm (average value of 2.76 mg·kg^−1^) and 10–0.0 cm (average value of 6.45 mg·kg^−1^).

### 3.3. Element Concentrations in the Movila Miresii Lake Sediment Core

**Figure 10:**
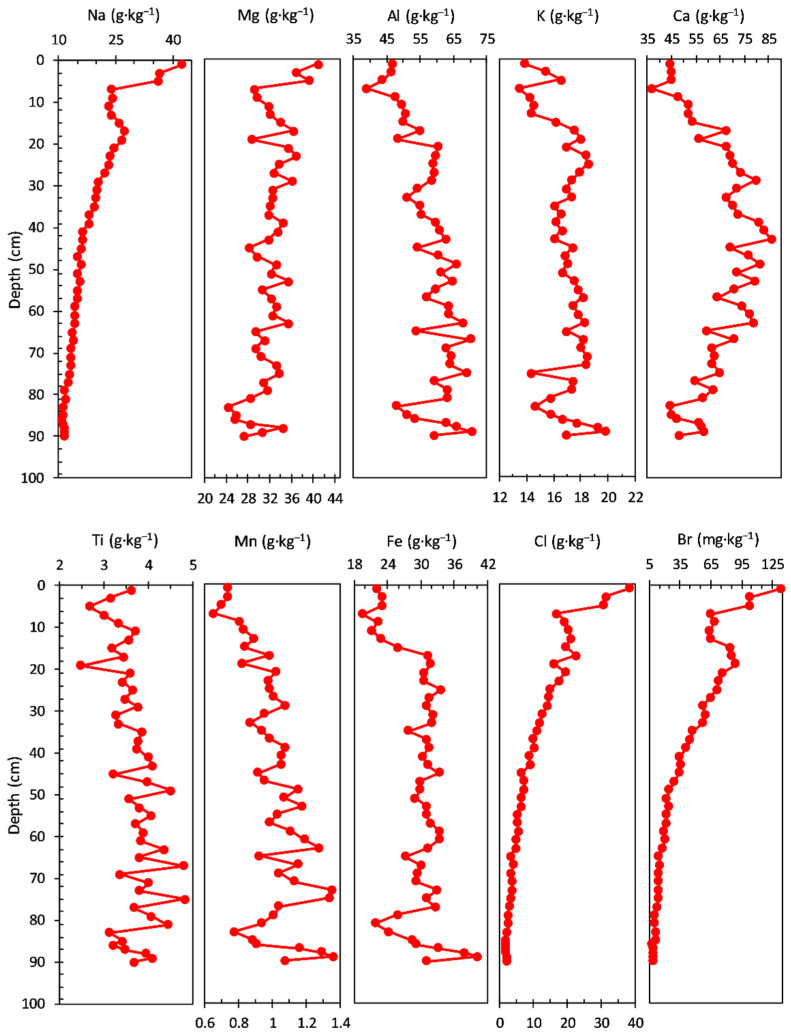
Vertical distribution of geochemical main elements (Na, Mg, Al, K, Ca, Ti, Mn, Fe, Cl, Br) in the Movila Miresii Lake sediment core (90 cm depth).

**Figure 11:**
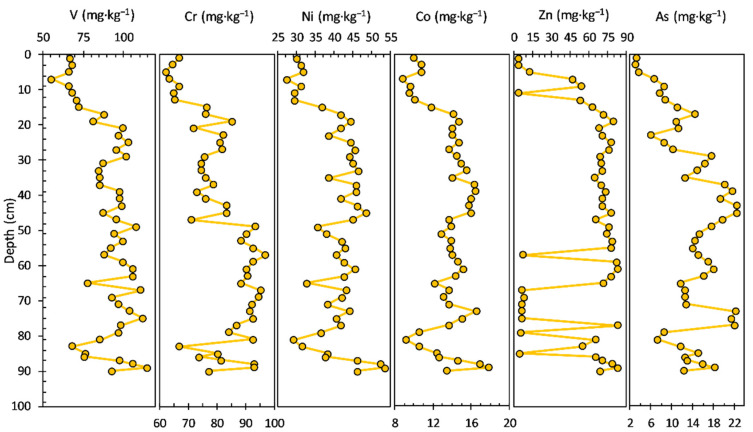
Vertical distribution of geochemical heavy metals (V, Cr, Ni, Co, Zn, As) in the Movila Miresii Lake sediment core (90 cm depth).

**Figure 12:**
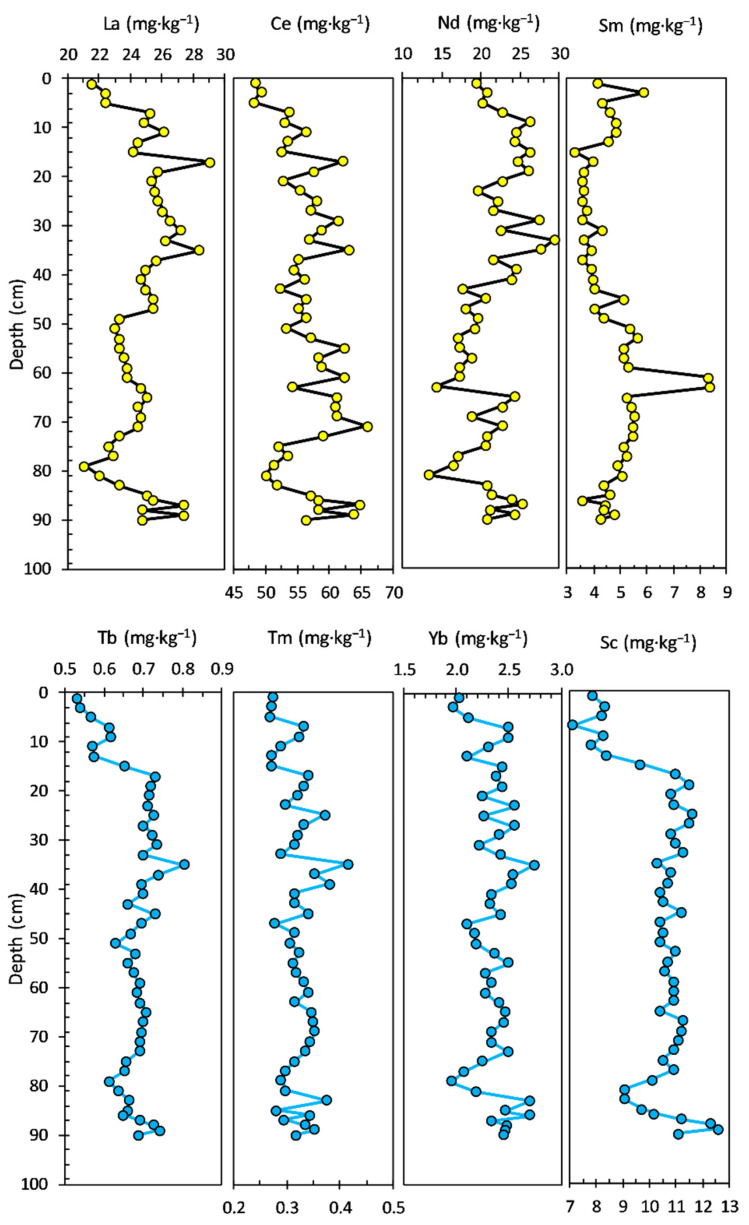
Vertical distribution of geochemical light rare earth elements—LREE (La, Ce, Nd, Sm) and heavy rare earth elements—HREE (Tb, Tm, Yb, Sc) in the Movila Miresii Lake sediment core (90 cm depth).

**Figure 13:**
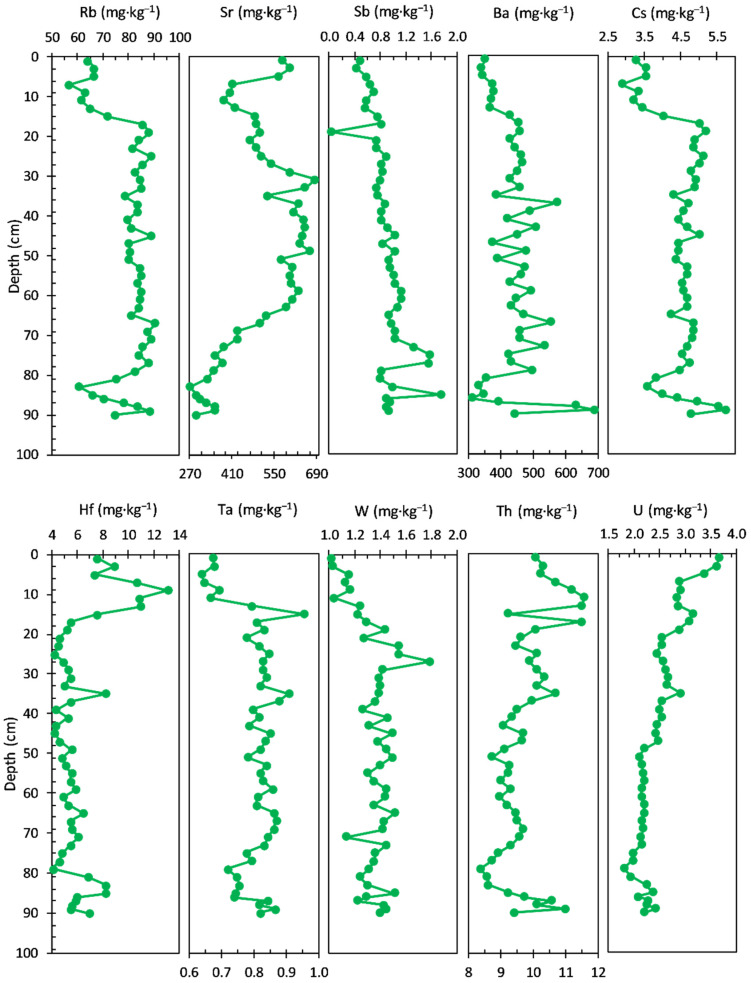
Vertical distribution of geochemical trace elements (Rb, Sr, Sb, Ba, Cs, Hf, Ta, W, Th, U) in the Movila Miresii Lake sediment core (90 cm depth).

The concentration profiles for main elements, heavy metals (HM), light rare earth elements (LREE), heavy rare earth elements (HREE), and trace elements (TE) are presented in Figure 10, Figure 11, Figure 12 and Figure 13. The results of the analysis did not show a distinct pattern for most of them; some of them occur in the smallest amount in the upper part of the sediment core, down to around 10 cm, and then occur in much higher concentrations below this depth. Other elements show an opposite trend, occurring in much higher amounts in the upper layer, with their highest concentration under this level.

The concentrations of the main elements measured in the sediment core are shown in Figure 10. It can be noticed that most of the analysed elements do not follow a distinct pattern, except for Na, Cl, and Br.

The concentration profiles of Na (ranged from 11.20 to 42.40 g·kg^−1^, with an average value of 18.45 g·kg^−1^), Cl (ranged from 1.75 to 38.40 g·kg^−1^, with an average value of 10.34 g·kg^−1^), and Br (ranged from 8.57 to 134.00 mg·kg^−1^, with an average value of 40.78 mg·kg^−1^) show a gradual increase in concentration structured on the depth, from the bottom to the surface, of the sediment core: 90–17 cm (Na—average value of 16.21 g·kg^−1^; Cl—average value of 7.45 g·kg^−1^; Br—average value of 31.77 mg·kg^−1^), 15–7 cm (Na—average value of 24.38 g·kg^−1^; Cl—average value of 19.50 g·kg^−1^; Br—average value of 69.36 mg·kg^−1^), and 7–0 cm (Na—average value of 34.90 g·kg^−1^; Cl—average value of 29.45 g·kg^−1^; Br—average value of 101.20 mg·kg^−1^).

On the contrary, Mg (ranged from 24.40 to 40.80 g·kg^−1^, with an average value of 32.04 g·kg^−1^), Ca (ranged from 36.80 to 86.20 g·kg^−1^, with an average value of 63.56 g·kg^−1^), and Ti (ranged from 2.48 to 4.82 g·kg^−1^, with an average value of 3.68 g·kg^−1^) concentrations do not show a specific pattern.

Concentration of Al (ranged from 39.00 to 70.50 g·kg^−1^, with an average value of 57.69 g·kg^−1^) and K (ranged from 13.40 to 19.80 g·kg^−1^, with an average value of 16.82 g·kg^−1^) share a similar pattern, except for the uppermost layer of sediment core. Mn (ranged from 0.66 to 1.36 g·kg^−1^, with an average value of 1.01 g·kg^−1^) and Fe (ranged from 19.50 to 40.20 g·kg^−1^, with an average value of 29.41 g·kg^−1^) also present a similar trend of concentration profile.

The concentration profiles of heavy metals (V, Cr, Ni, Co, Zn, and As) measured in the sediment core are shown in Figure 11. Zn and As display a different pattern relative to the rest of the analysed HM (V, Cr, Ni, and Co show almost a similar trend in the depth of the sediment core, especially in the upper sediment layers above 35 cm). V (ranged from 55.50 to 115.00 mg·kg^−1^, with an average value of 90.23 mg·kg^−1^), Cr (ranged from 62.30 to 97.20 mg·kg^−1^, with an average value of 81.33 mg·kg^−1^), Ni (ranged from 27.20 to 53.30 mg·kg^−1^, with an average value of 40.23 mg·kg^−1^), and Co (ranged from 8.75 to 17.80 mg·kg^−1^, with an average value of 13.48 mg·kg^−1^) concentrations show the highest variations in the half bottom of the sediment core.

The concentration profile of Zn (ranged from 3.46 to 82.80 mg·kg^−1^, with an average value of 54.34 mg·kg^−1^) displays significant variations in the upper (13–0.0 cm) and bottom (90–55 cm) sediment layers. The As concentration (ranged from 2.88 to 22.50 mg·kg^−1^, with an average value of 13.68 mg·kg^−1^) also does not present a specific pattern.

The vertical distribution of the LREEs and HREEs concentrations in the sediment core is illustrated in Figure 12. All four LREEs (La—ranged from 21.10 to 29.10 mg·kg^−1^, with an average value of 24.73 mg·kg^−1^; Ce—ranged from 48.10 to 65.80 mg·kg^−1^, with an average value of 56.52 mg·kg^−1^; Nd—ranged from 13.40 to 29.30 mg·kg^−1^, with an average value of 21.41 mg·kg^−1^; Sm—ranged from 3.26 to 8.34 mg·kg^−1^, with an average value of 4.66 mg·kg^−1^) do not show a specific pattern or a similar concentration profile, with the main differences noticed for La, Ce, and Nd in the upper 20 cm sediment layer.

The concentration profiles of HREEs (Tb—ranged from 0.53 to 0.80 mg·kg^−1^, with an average value of 0.68 mg·kg^−1^; Tm—ranged from 0.27 to 0.42 mg·kg^−1^, with an average value of 0.32 mg·kg^−1^; Yb—ranged from 1.95 to 2.74 mg·kg^−1^, with an average value of 2.35 mg·kg^−1^; Sc—ranged from 7.13 to 12.6 mg·kg^−1^, with an average value of 10.38 mg·kg^−1^) suggest the same general pattern, with a decrease in the upper 20 cm sediment layer.

The concentration profiles of the trace elements measured in the sediment core are shown in Figure 13.

Sr (ranged from 271.00 to 686.00 mg·kg^−1^, with an average value of 499.10 mg·kg^−1^), Th (ranged from 7.89 to 11.1 mg·kg^−1^, with an average value of 9.25 mg·kg^−1^), and U (ranged from 1.80 to 3.67 mg·kg^−1^, with an average value of 2.47 mg·kg^−1^) display a different trend relative to the rest of analysed elements (gradually increase from the bottom to the surface of the sediment core).

In addition, the other elements display the same trend. The most similarities are noticed between Rb (ranged from 56.90 to 90.60 mg·kg^−1^, with an average value of 79.53 mg·kg^−1^) and Cs (ranged from 2.87 to 5.73 mg·kg^−1^, with an average value of 4.46 mg·kg^−1^). On the contrary, Ba (ranged from 312.00 to 689.00 mg·kg^−1^, with an average value of 440.35 mg·kg^−1^), W (ranged from 1.02 to 1.79 mg·kg^−1^, with an average value of 1.35 mg·kg^−1^), and Sb (ranged from 0.04 to 1.75 mg·kg^−1^, with an average value of 0.89 mg·kg^−1^) also follow a relatively similar pattern, except for the bottom half of the sediment core.

The concentration profiles of Hf (ranged from 4.11 to 13.10 mg·kg^−1^, with an average value of 6.19 mg·kg^−1^) and Ta (ranged from 0.64 to 0.96 mg·kg^−1^, with an average value of 0.80 mg·kg^−1^) have an almost similar pattern, except for the uppermost layer of the sediment core (above 20 cm depth).

### 3.4. Determination of Geochemical Background Value

The geochemical background value (GBV) was quantified using the elements’ concentration in the 10 cm area at the base of the sediment sample core [5]. The M2MAD or median and MAD coefficient method [Md±2MAD] based on the median value (Md) and median absolute deviation (MAD) was used to determine GBV [76,77,78,79]. In the first stage of the analysis, the values of the minimum, mean, maximum, median (Md), and standard deviation (SD) were calculated for the lakes’ bottom sediment core (Table 2, Table 3 and Table 4). The analysed data sets were tested for normality (the Shapiro–Wilk test, the Anderson–Darling test, the Lilliefors test, and the Jarque–Bera test). The Grubbs test for outliers (alternative hypothesis: two-sides; significance level: 5%, alpha 0.005) showed that there were 15 outlier observations in the Amara Lake dataset (Table 2), 17 outlier observations in the Caineni Lake dataset (Table 3), and 5 outlier observations in the Movila Miresii Lake dataset (Table 4).

Among the analysed elements from lake Amara’s bottom sediment core, most were normally distributed, except for Na, Al, Cl, Ca, Fe, Nd, Sb, and W, which were log-normally distributed (the distributions were right-skewed, mean values were higher than median ones). The dataset for Lake Caineni’s bottom sediment reveals that most of the concentration values were log-normally distributed, except for Mg, Cl, Ca, Ti, Mn, and V (normally distributed). In the Lake Movila Miresii analysed dataset, Al, Ca, Ni, Zn, Ce, Rb, and U were log-normally distributed, and the rest of the elements were normally distributed. The calculated GBV values for each lake’s elemental composition dataset are presented in Table 5.

### 3.5. Contamination Assessment

#### 3.5.1. Assessment of Pollution Indices from the Amara Lake Sediment Core

**Figure 14:**
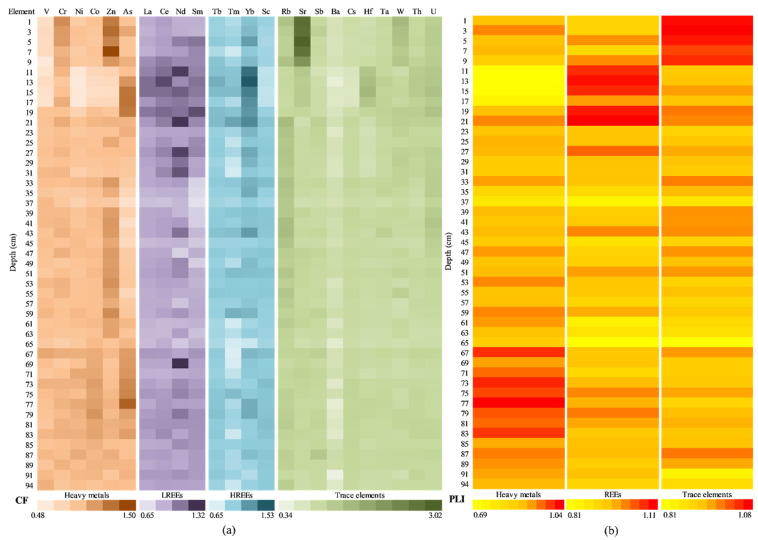
Contamination factors (CFs) (**a**) and pollution load indices (PLIs) (**b**) calculated from the Amara Lake sediment core (94 cm depth).

Figure 14 presents the contamination degree assessment of the individual potential contaminants (divided in HMs, LREEs, HREEs, and TEs), by CF and PLI, at the depth level of the sediment core. For most of the sediment core sections analysed, the contamination factor values (Figure 14a) were smaller than 1, indicating a low degree of contamination: HMs (ranged from 0.48 to 1.50, average value 0.91), LREEs (ranged from 0.65 to 1.32, average value 0.96), HREEs (ranged from 0.65 to 1.53, average value 0.96), and TEs (ranged from 0.34 to 3.02, average value 0.99).

The majority of the analysed HMs showed contamination factor values higher than 1 (moderate degree of contamination), mostly in the upper (above 21 cm) and bottom (below 65 cm) sections of the sediment core. V and Zn present a more particular display, with V exceeding 1 only in one section core (67 cm) and Zn having CF values higher than 1 in the middle sections of the sediment (39–63 cm) also.

The CFs of Cr, Zn, and As show moderate contamination in the upper sections, which can be due to the natural weathering of the minerals (a known source of Zn) [80], the use of Pb-As insecticides and arsenic-based herbicides [34,81,82,83] on agricultural land in neighboring areas, or atmospheric inputs (precipitation of aerosol particles released by quarrying activities) [84]. The most important sources of anthropogenic zinc in the soil also include coal and bottom fly ash, as well as the use of commercial products, such as fertilizers and wood preservatives, that contain zinc [85].

The CF values higher than that of Cr in the upper sections also suggest the input of natural weathering/sources (111.26 mg·kg^−1^ average value relative to 112.40 mg·kg^−1^ calculated GBV_Cr_). For LREEs, CF values higher than 1 were present, especially in the 5–31 cm and 73–85 cm depth ranges. The highest average CF values of the entire sediment core were obtained for Ce (0.97) and Nd (1.00). HREEs contamination factors higher than 1 have been registered, mostly in the upper (above 23 cm), middle (37–43 cm), and bottom (below 71 cm) sections of the sediment core; the average values of CFs of the entire sediment core were close to 1 (except for Nd, with an average value of 1.00): La (0.94), Ce (0.97), and Sm (0.93). Chen et al. [86] considered the REE migration processes from the uplands to the lowlands as presenting enrichments in terminal paddy fields; the higher contents inside rock cavities could be attributed to a lower loss by leaching or an increased precipitation inside cavities, which are semi-enclosed systems flooded during high waters and dried by evaporation in the dry seasons [87]. The accumulation or retention seemed to be more intense in the surface sediments of rock cavities and could be related to retention during the development of coatings and biofilms, as previously observed for other trace elements [88]. For TEs, contamination factor average values lower than 1 on the entire depth of the sediment core ranged from 0.70 (Ba) to 0.98 (Th), indicating a low degree of contamination; the lowest CFs values were obtained for Ba (ranged from 0.34 to 0.90). A moderate degree of contamination was noticed for the rest of the TEs (except for Ba) in different sections of the sediment core. Sr registered the highest CF values (ranged from 2.18–3.02) in the 1–9 cm depth sections.

Figure 14b shows the results from the PLI analysis, with values ranging from 0.69 to 1.04 for HMs (mean 0.90), 0.81 to 1.11 for REEs (mean 0.96), and 0.81 to 1.08 for TEs (mean 0.94). PLI values very close to 1 indicate a slightly progressive deterioration of the environment.

**Figure 15:**
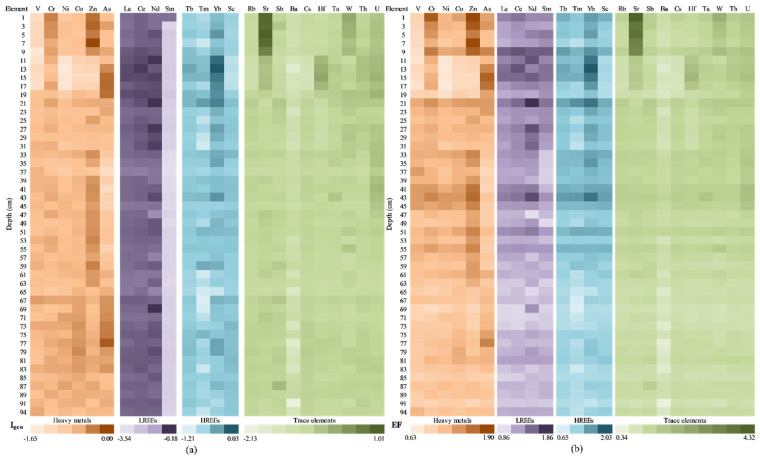
Geoaccumulation indices (I_geo,s_) (**a**) and enrichment factors (EFs) (**b**) calculated from the Amara Lake sediment core (94 cm depth).

The results obtained for the geoaccumulation index (I_geo_) are presented in Figure 15a. The I_geo_ values indicate that the sediment was uncontaminated (I_geo_ ≤ 0) for HMs, REEs, and for most of the investigated TEs. None to moderate contamination (I_geo_ = 0 ÷ 1) and moderate contamination (I_geo_ = 1 ÷ 2) were found for Sr in the uppermost sections of the sediment core (above 10 cm), indicating the contributions from anthropogenic pollution sources. The influence of anthropogenic sources on the investigated elements in the sediment core sections samples was also assessed by determining the EF values (Figure 15b). The EF is a frequently used parameter for assessing the impact of anthropogenic activities in sediments by distinguishing between natural and human sources of a single element [89,90].

Accordingly, EF values less than one suggest that the element mostly came from natural processes or crustal materials, whereas EF values greater than one indicate that the sources are more likely to be anthropogenic [91]. When the EF is normalized against the tested element’s background value, the anthropogenic contamination prognosis becomes better [92,93]. For most sections analysed, the obtained values of EF less than 1.5 were obtained for the investigated elements, indicating their most probable natural origin/sources, with some exceptions observed for HMs (Cr, Zn, and As), REEs, and TEs (except for Rb, Ba, and Cs).

EF values suggesting a minor anthropogenic modification (EF = 1.5 ÷ 3) where obtained for Cr (1.77) and As (1.55 ÷ 1.90) in the upper sediment core sections and for Zn 1.55 ÷ 1.90) in the upper (above 10 cm) and middle (41–55 cm) sections. For both LREEs (1.52 ÷ 1.86) and HREEs (1.50 ÷ 2.03), the obtained EF values (1.5 ÷ 3) were registered in the 9–43 cm depth level. Moderate anthropogenic modification (EF = 3 ÷ 5) was calculated only for Sr (3.36 ÷ 4.32) in the uppermost layer of the sediment (above 10 cm). 

#### 3.5.2. Assessment of Pollution Indices from the Caineni Lake Sediment Core

**Figure 16:**
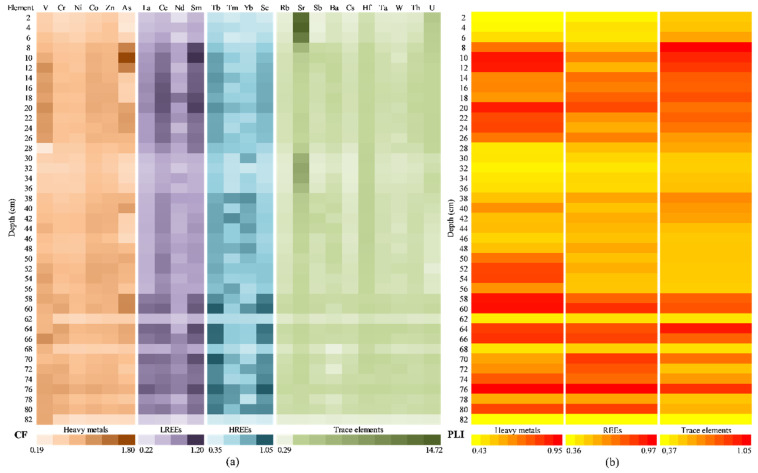
Contamination factors (CFs) (**a**) and pollution load indices (PLIs) (**b**) calculated from the Caineni Lake sediment core (82 cm depth).

Figure 16 presents the contamination level assessment for the potential contaminants of Caineni Lake (separated as HMs, LREEs, HREEs, and TEs), using the CF and PLI indices. For contamination factor (Figure 16a), most of the analysed samples (sediment core sections) recorded values smaller than 1, which indicates a low contamination degree: HMs (ranged from 0.20 to 1.80, with the average value of 0.77), LREEs (ranged from 0.22 to 1.20 and the average value 0.78), HREEs (ranged from 0.35 to 1.05, average value 0.72), and TEs (ranged from 0.29 to 14.72, average value 1.03).

The most analysed HMs showed that contamination factor values were lower than 1 (low degree of contamination) with two exceptions: V in the sections of the sediment core from 12 cm up to 26 cm and As in the areas of the sediment core from 8 cm up to 12 cm and 20 cm up to 24 cm. In addition, V recorded values higher than 1 in the sediment core sections of 54 cm, 66 cm, and 76 cm, while As recorded values higher than 1 in the sediment core sections of 40 cm, 58 cm, and 60 cm. Furthermore, samples collected from Caineni Lake at 64 cm and 76 cm present values higher than 1 for Cr. The CF levels for V, As, and Cr can be due to natural (their presence in Earth’s crust and seawater/saltwater) [94,95,96] or anthropogenic (agricultural treatments) sources [97].

In the case of LREEs, the CF values were lower than 1 for most of the analysed sediment core sections, except for the samples 8–10 cm (for Sm); 14–20 cm (for Ce and Sm); 60 cm, 64 cm, 66 cm, and 70 cm (for Sm); and 76 cm (for La and Sm). Even so, the maximum recorded values were close to 1 (i.e., La 1.04, Ce 1.07, Nd 0.95, and Sm 1.20), and the mean values were similar (i.e., La 0.72, Ce 0.86, Nd 0.68, and Sm 0.87). In the case of HREEs, the CF values were close to 1, with a few exceptions: 60 cm (Tb and Sc); 64 cm, 66 cm, and 70 cm (Tb); and 76 cm (Tb and Sc). All these samples recorded values in the range of 1.01 to 1.05. All these rare-earth elements are widely distributed in the Earth’s crust [98], but do not occur in pure form naturally [99]. However, Jiang et al. [100] consider that the REEs distribution pattern in natural water bodies and sediments is influenced by both natural and human activities, and Sanders et al. [101] reveal that high REEs contents in estuarine sediments can be related to fertilizer usage or industry.

For TEs, the values for CF were lower than 1 on the entire depth of the sediment core for Rb (in the range of 0.38–0.99, with average value of 0.69) and Cs (in the range of 0.36–0.98, with average value of 0.67). At the same time, for Sb (0.31–1.13, average value 0.75), Ba (0.29–1.09, average value 0.81), Hf (0.40–1.69, average value 1.17), Ta (0.38–1.07, average value 0.80), W (0.34–1.09, average value 0.74), and Th (0.37–1.05, average value 0.84), the CFs values were close to 1, indicating a low or moderate degree of contamination. The samples collected at 10–20 cm depth recorded values higher than 1 for Ba, Hf, and Th. In the case of Sr, the samples collected at 2–6 cm were characterised by a very high degree of contamination (11.85–14.72), as well as the samples collected at 32 cm and 34 cm (6.36–6.39). The samples collected at 8 cm, 30 cm, and 36 cm were characterised by a considerable degree of contamination (4.43–5.90). The other samples were included in the categories of sediments with low or moderate degrees of contamination. Mirzoyeva et al. [102] reported that the Sr content in sediments collected from salt lakes depends on the salinity level of the lakes’ water.

Figure 16b shows the results from PLI analysis, with values ranging from 0.43 to 0.95 for HMs (average value 0.75), 0.36 to 0.97 for REEs (average value 0.74), and 0.37 to 1.05 for TEs (average value 0.87). PLI value lower than 1 indicate only baseline pollutants of the environment [34].

**Figure 17:**
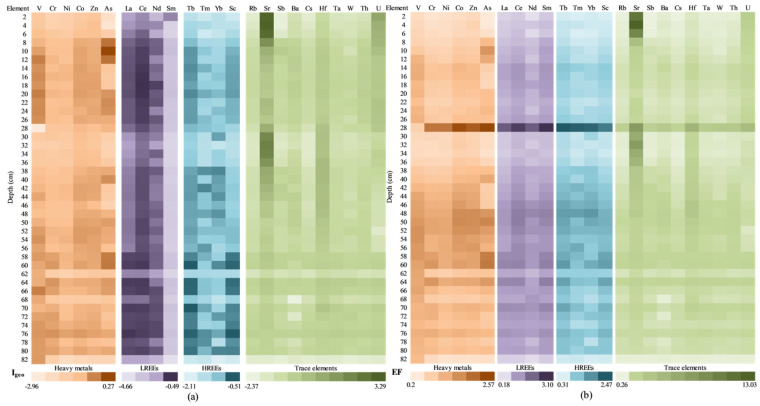
Geoaccumulation indices (I_geo,s_) (**a**) and enrichment factors (EFs) (**b**) calculated from the Caineni Lake sediment core (82 cm depth).

The obtained results for the geoaccumulation index (I_geo_) and enrichment factors (EFs) are presented in Figure 17a. In the case of I_geo_, calculated for all categories (HMs, LREEs, HREEs, and TEs), the obtained values highlight that the sediment samples were uncontaminated (I_geo_ ≤ 0), except for U and Sr, which establishes the none to moderate contamination up to heavy contamination of the samples collected at 2–10 cm, as well as 30–36 cm. Taking into account the fact that Caineni Lake does not have any spring or surface water sources (which involves a low sedimentation rate), it is possible that the U and Sr in the segment of 2–10 cm are the results of the Chernobyl accident, as was highlighted by Mirzoyeva et al. also [102].

The effects of anthropic activity on the analysed elements in the sediment core sections were assessed using the enrichment factor (Figure 17b). As aforementioned, the EF represents the parameter used for assessing the human impact on the environment, and it distinguishes between natural and human sources of each element [89,90].

The maximum EF value for all elements was higher than 1.5, and these maximum values were recorded in the same sample (i.e., 28 cm depth, except for V and Sr); this fact highlights a moderate influence of the anthropogenic activity. Samples collected at 48–52 cm depth present values close to 1.5 for all elements.

The lowest values for the EF index were recorded in the samples collected at 2 cm (in the case of V, Cr, As, La, Nd, Tb, Tm, Sc, Rb, Sb, and Cs) or 82 cm (in the case of Ni, Co, Zn, Ce, Sm, Yb, Sr, Hf, Ta, W, Th, and U). A severe (EF = 5 ÷ 10) and/or very severe anthropogenic modification (EF > 10) was reported only for Sr for 2–6 cm sediment core sections.

#### 3.5.3. Assessment of Pollution Indices from the Movila Miresii Lake Sediment Core

**Figure 18:**
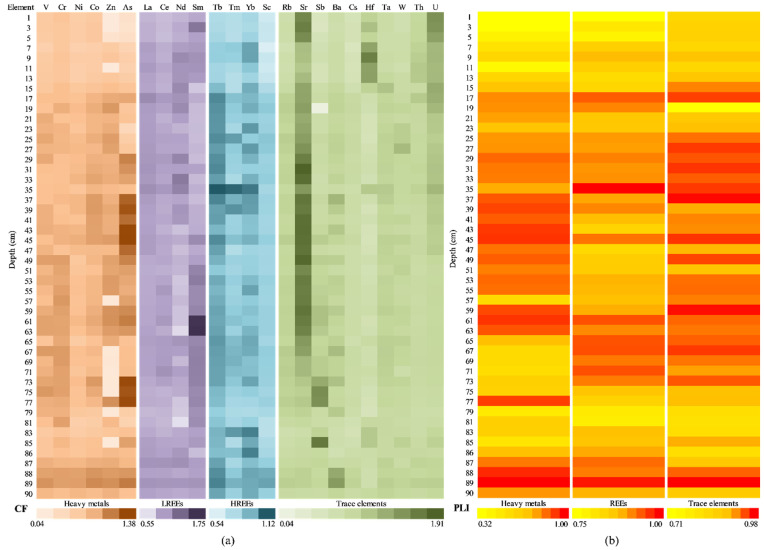
Contamination factors (CFs) (**a**) and pollution load indices (PLIs) (**b**) calculated from the Movila Miresii Lake sediment core (90 cm depth).

Figure 18 presents the contamination level assessment for the potential contaminants of Movila Miresii Lake (separated as HMs, LREEs, HREEs, and TEs), using the CF and PLI indices. For contamination factor (Figure 18a), most of the analysed samples (sediment core sections) recorded values smaller than 1, which indicates a low contamination degree: HMs (ranged from 0.44 to 1.38, with the average value of 0.77), LREEs (ranged from 0.55 to 1.75 and the average value 0.91), HREEs (ranged from 0.54 to 1.12, average value 0.87), and TEs (ranged from 0.04 to 1.91, average value 0.93).

The most analysed HMs showed that contamination factor values were lower than 1 (low degree of contamination), with three exceptions: Co in the section of the sediment core of 89 cm; Zn in the areas of the sediment core of 59 cm, 61 cm, 77 cm, and 89 cm; and As in the samples corresponding to a depth of 29 cm, 31 cm, 37–49 cm, 59 cm, 61 cm, 73–77 cm, and 89 cm. The CF levels for As and Zn could be due to natural (their presence in Earth’s crust and seawater/saltwater) [96] or anthropogenic (chemical solution used in agriculture: pesticides and fertilizer) sources [103].

In the case of LREEs, the CF values were lower than 1 for most of the analysed sediment core sections, except for the samples: 17 cm, 31 cm, 35 cm, 87 cm, and 89 cm (for La); 71 cm and 87 cm (for Ce); 9–19 cm, 29 cm, 33–35 cm, 39 cm67 cm, and 87 cm (for Nd); and 3 cm, 9–11 cm, 45 cm, 51–81 cm, and 89 cm (for Sm). Even so, the maximum recorded values were close to 1 (i.e., La 1.07, Ce 1.02, Nd 1.20, and Sm 1.75), and the mean values were quite similar (i.e., La 0.91, Ce 0.88, Nd 0.88, and Sm 0.98). In the case of HREEs, the CF values were close to 1, with a few exceptions: 17–19 cm, 25 cm, 29–31 cm, 35–37 cm, 45 cm, and 88–89 cm (Tb); 35 cm and 39 cm (Tm); and 35 cm, 83 cm, and 86 cm (Yb). All these samples recorded values in the range of 0.54–1.12. As abovementioned, these rare-earth elements are present in the Earth’s crust [95], not in pure form [96], and their distribution depends on natural and human activities (i.e., agriculture and industry) [100,102].

For TEs, the values for CF were lower than 1 on the entire depth of the sediment core for Cs (in the range of 0.48–0.97, with average value of 0.75). At the same time, for Sb (0.04–1.73, average value 0.88), Ba (0.65–1.45, average value 0.92), Hf (0.52–1.67, average value 0.79), Ta (0.74–1.11, average value 0.93), W (0.67–1.17, average value 0.88), and Th (0.75–1.05, average value 0.88), the CF values were close to 1, indicating a low or moderate degree of contamination. The samples collected at 1–47 cm depth recorded values higher than 1 for Sr and U, which were characterised by a moderate degree of contamination (1÷3), as well as the samples collected at 49–73 cm (just for Sr). The possible cause for this moderate contamination can be the Chernobyl accident, and it depends on the water salinity [102].

Figure 18b shows the results from the PLI analysis, with values ranging from 0.32 to 1.00 for HMs (average value 0.73), 0.75 to 1.00 for REEs (average value 0.88), and 0.71 to 0.98 for TEs (average value 0.90). These values indicate that just baseline pollutants are present in the environment [34].

**Figure 19:**
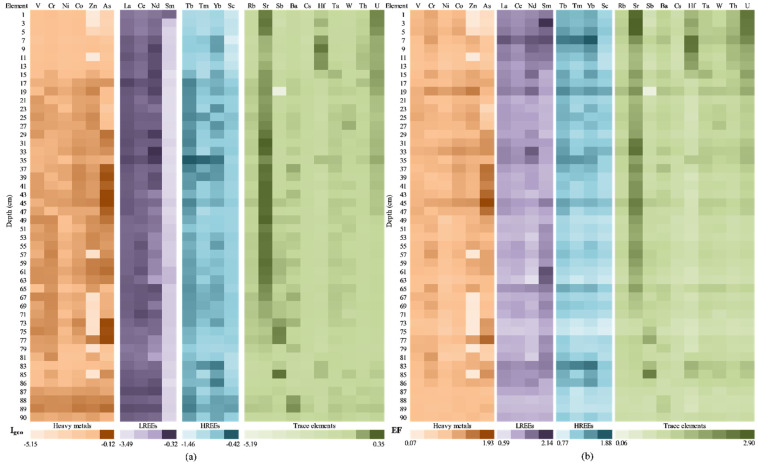
Geoaccumulation indices (I_geo,s_) (**a**) and enrichment factors (EFs) (**b**) calculated from the Movila Miresii Lake sediment core (90 cm depth).

The obtained results for the geoaccumulation index (I_geo_) and the enrichment factors (EFs) are presented in Figure 19. In the case of I_geo_ (Figure 19a), calculated for all categories (HMs, LREEs, HREEs, and TEs), the obtained values highlight that the sediment samples were uncontaminated (I_geo_ ≤ 0), except for Sr (samples collected at 1–5 cm, 27–33 cm, and 37–63 cm depth), Sb (for samples 75–77 cm and 85 cm), Hf (9 cm depth), and U (1 cm depth), which establish the none to moderate contamination. Even if the CF values are close to 0, Movila Miresii should be constantly monitored in order to establish the measurements that need to be taken.

The enrichment factor (Figure 19b) allows assessing the effects of anthropic activity on the chemical content of sediments, as well as distinguishing between natural and human sources of each element [89,90]. The maximum EF value for La, Ce, Nd, Tb, Tm, Yb, Hf, and Th were recorded in the same sample (i.e., 7 cm depth); this fact highlights a minor influence of the anthropogenic activity. Corroborated with the CF values—for Sr and U—the 1–19 cm core section was exposed to low-level pollution, but for a long time. The lowest values for the EF index were recorded in the samples collected at 75 cm (in the case of V, Cr, Ni, Zn, La, Ce, Tb, Tm, Sc, Rb, Ba, Cs, Hf, Ta, Th, and U).

### 3.6. Correlation Analysis and Principal Component Analysis

**Figure 20:**
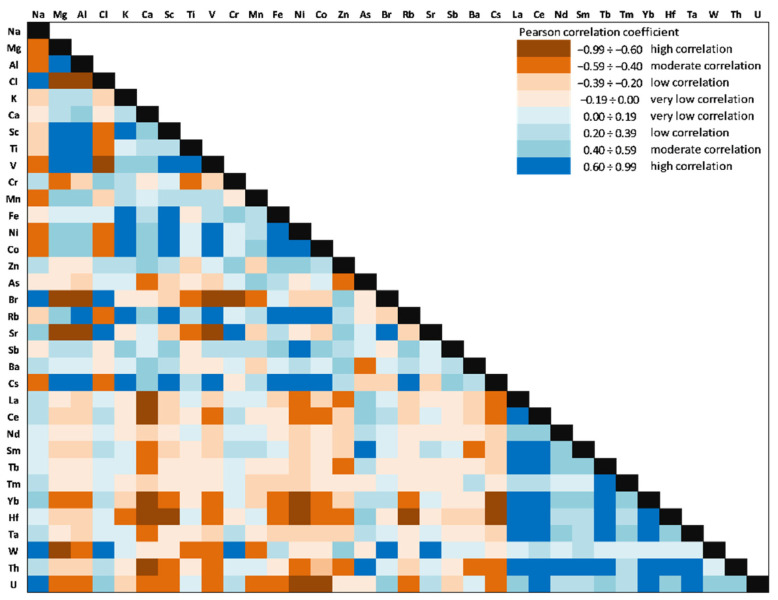
Pearson correlation map based on coefficient matrix for the relationship between the element concentrations in the Amara Lake sediment core.

The correlations between the 34 investigated elements (Person correlation coefficients) in the Amara Lake sediment core are presented in Figure 20. Significant positive correlations (significance level alpha = 0.05) for V with Ni (0.68), Co (0.65), Mn (0.39), Rb (0.75), and Cs (0.78) might indicate that they originate from the same sources and have similar transformation and migration processes.

Ni was significantly correlated with Co (0.89), Rb (0.85), and Cs (0.90). Zn was significantly negatively correlated with all REEs, except for Tm (−0.03). As was positively correlated with all LREEs (La: 0.56; Ce: 0.49; Nd: 0.30, and Sm: 0.69) and some of TEs (Hf: 0.44; Ta: 0.32, and Th: 0.60). As a major component of clay minerals, Al showed significantly positive correlations with V (0.95), Ni (0.55), and Co (0.57) as well, thus suggesting a natural origin of these HMs. High values of the Pearson coefficients (positive correlation) were obtained between LREEs (0.56÷0.91) and HREEs (0.50÷0.71), except for Sc, and significantly positive correlations were obtained for Sc with HMs (V: 0.77; Ni: 0.87, and Co: 0.87), Rb (0.95), and Cs (0.97).

**Figure 21:**
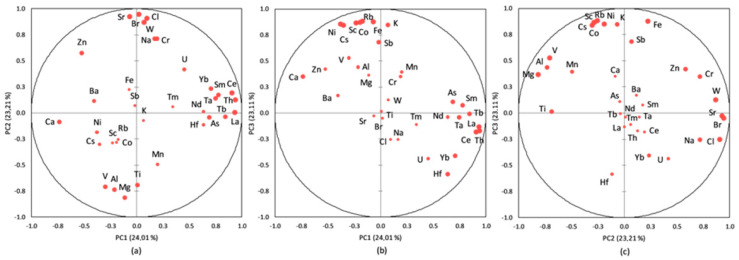
PCA box plots, PC1 versus PC2 (**a**), PC1 versus PC3 (**b**), and PC2 versus PC3 (**c**) obtained for quantified elements in sediment sample of Amara Lake.

The PCA performed on the database aimed to identify the most important sources for HMs, REEs, and TEs in the sediment core of Amara Lake. The results from the PCA applied to the database are presented in Figure 21 and Table 6. The first three principal components with eigenvalues greater than 1.00 were selected, with 70.33% of the variance explained. The measure of sampling adequacy by KMO statistics provided the value of 0.71, while the significance of Bartlett’s test of sphericity was less than 0.001, which led to the conclusion that the database size was suitable for evaluation by the PCA. As presented in Table 6, the first three principal components loadings were classified as strong (loadings values > 0.60) and moderate (loading values 0.60 ÷ 0.30).

Figure 21 presents the PCA box plots (correlations between variables and the principal components after Varimax rotation), with the loading of PC1 versus PC2, PC1 versus PC3, and PC2 versus PC3 from the PCA applied to the investigated dataset.

PC1 had strong loadings of most LREEs (except for Tm, with moderate loading), HREEs (except for Sr), Ta, Hf, Th, and As and moderate loadings of U, corresponding to the pedological characteristics of the area. PC2 highlights strong loadings for Na, Cl, Br, Sr, Zn, Cr, and W and moderate loadings for U and Yb; this principal component can be attributed to a mixed source of salts (due to the lack of a constant supply of freshwater and through evaporation during dry periods) and dry or wet atmospheric deposition. The moderate/strong loadings for Zn (0.58), Cr (0.72), and W (0.87) indicate an anthropogenic input; other studies [34,104,105,106] indicated Zn, W, and Cr as tracers of anthropogenic factor (pollution from the urban roads dust). PC3 with strong loadings of K, Sc, Cs, Co, Fe, Ni, Rb, and Sb and moderate loadings of Al, Ca, Mg, Mn, Zn, V, and Cr can be attributed to leaching from soil surface/denudation, rock weathering and mixed anthropogenic input (e.g., agricultural runoff, wastewater discharges).

**Figure 22:**
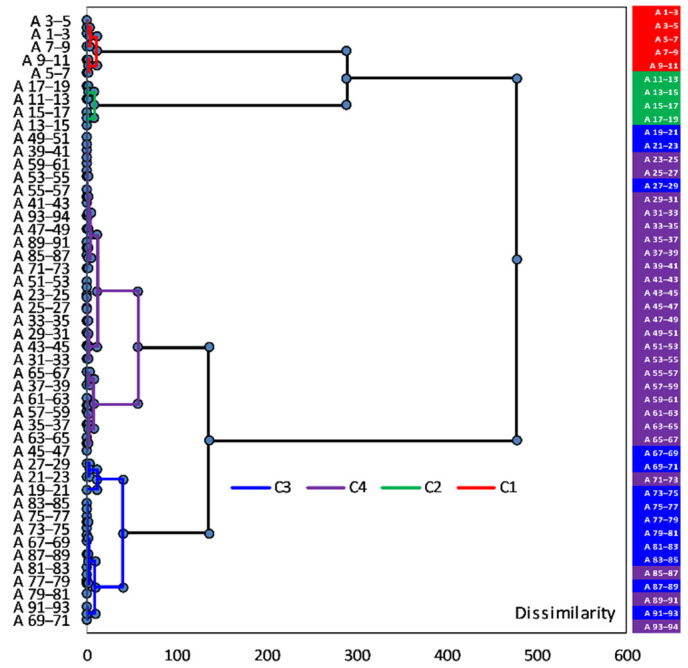
Dendrogram showing the clustering and vertical distribution (on depth) for the sediment sections of Amara Lake.

PCA analysis was followed by AHC applied to the factors/principal components score of the sediment core sections, i.e., the 47 sections (from A 1–3 cm to A 93–94 cm) in which investigated elements were analysed in order to group similar sections (vertical variability). The AHC results were rendered as a dendrogram (Figure 22), where all the sediment sections were clustered in four statistically significant groups/clusters—inertia decomposition for the optimal classification: within-cluster 18.03% and between-clusters 81.97%, H(k − 1)–H(k) at 12.31, the Hartigan index (H) of a clustering with k clusters and a clustering with (k − 1) clusters.

The four clusters obtained, C1, C2, C3, and C4 (Figure 22), can offer a better view of sediment core stratification based on the statistical approach to the dataset. A clear differentiation of C1 (sections from A 1–3 cm to A 9–11 cm) and C2 (sections from A 11–13 cm to A 17–19 cm) clusters, which includes sections from the upper layers of the sediment core, can be observed. C3 and C4 clusters’ relatively lower homogeneity (within-cluster variance 5.90 for C3 and 4.14 for C4) suggests certain elements’ migration or geochemical and weathering events that require further investigation.

**Figure 23:**
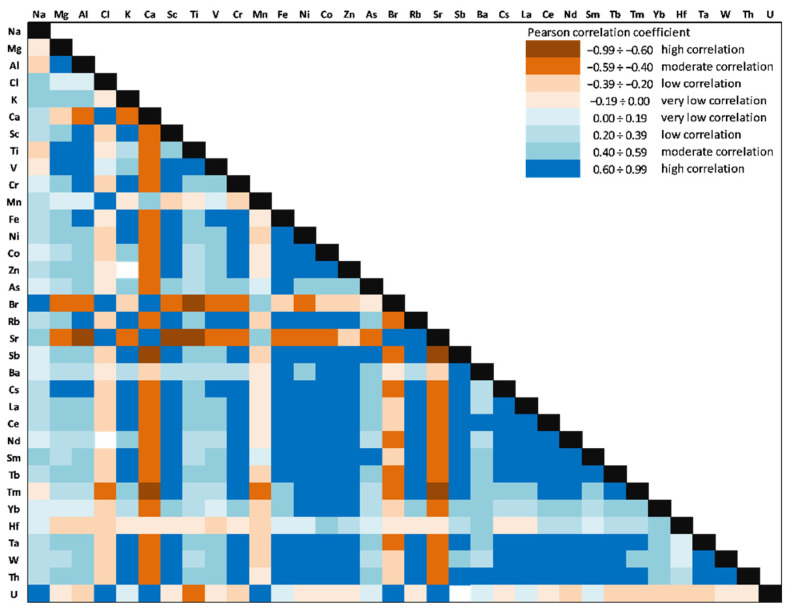
Pearson correlation map based on coefficient matrix for the relationship between the element concentrations in the Caineni Lake sediment core.

The correlations between investigated elements (Person correlation coefficients) in the Caineni Lake sediment core are presented in Figure 23.

Significantly positive correlations (significance level alpha = 0.05) were obtained for V with Al (0.99), Mg (0.94), Ti (0.88), Fe (0.65), Rb(0.64), and Cs(0.69), which might indicate that they originate from the same sources and have similar migration processes. Cr was significantly correlated with K (0.81, Sc (0.97), Ni (0.90), Co (0.71), Zn (0.71), Rb (0.97), Sb (0.75), and REEs (0.61÷0.90). For Fe, Ni, Co, and Zn, an almost similar pattern was obtained, with positive high correlation for Rb (0.70÷0.89), Sb (0.76÷0.81), Ba (0.58÷0.75), Cs (0.67÷0.83), REEs (0.42÷0.92), Ta (0.72÷0.88), W (0.63÷0.80), and Th (0.90÷0.94). Aluminium showed significantly positive correlations with Ti (0.91), V (0.99), Cr (0.60), Fe (0.62), Rb (0.63), Cs (0.67), and REEs (0.40÷0.56). High values of Pearson coefficients (positive correlation) were obtained between LREEs (0.65÷0.96) and HREEs (0.45÷0.81). Significantly positive correlations were obtained for Sc with HMs (0.62÷0.97), Sb (0.78), Cs (0.99), and LREEs (0.68÷0.96).

**Figure 24:**
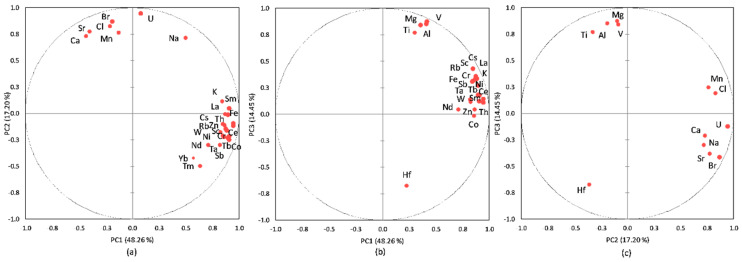
PCA box plots, PC1 versus PC2 (**a**), PC1 versus PC3 (**b**), and PC2 versus PC3 (**c**) obtained for quantified elements in sediment sample of Caineni Lake.

The results from the PCA applied to the Caineni Lake database are presented in Figure 24 and Table 7. The first three principal components with eigenvalues greater than 1.00 were selected, with 79.92% of the variance explained. The measure of sampling adequacy by KMO statistics provided the value of 0.78, while the significance of Bartlett’s test of sphericity was less than 0.001; the database size was suitable for evaluation by the PCA. As presented in Table 7, the first three principal components loadings were classified as strong (loadings values > 0.60) and moderate (loading values 0.60 ÷ 0.30).

Figure 24 presents the PCA box plots for the Caineni Lake dataset (correlations between variables and the principal components after Varimax rotation), with the loading of PC1 versus PC2, PC1 versus PC3, and PC2 versus PC3 from the PCA applied to the dataset.

PC1 reveals strong loadings for LREEs, HREEs, TEs (except for U and Hf), HMs (except for V), K, Sc, Fe, and Rb, corresponding to pedological characteristics of the area. PC2 highlights strong loadings for Na, Cl, Br, Sr, Ca, Mn, and U; this principal component can be attributed to a mixed source of salts, loess fingerprint, and dry or wet atmospheric deposition. PC3, with strong loadings of Al, Mg, Ti, and V, can be attributed to leaching from soil surface/denudation and rock weathering. PCs plots and the Pearson correlation map (Figure 23) also reveal strong negative loading and low correlations of Hf; this may suggest element sensitivity to the redox status of the sediment [107,108,109].

**Figure 25:**
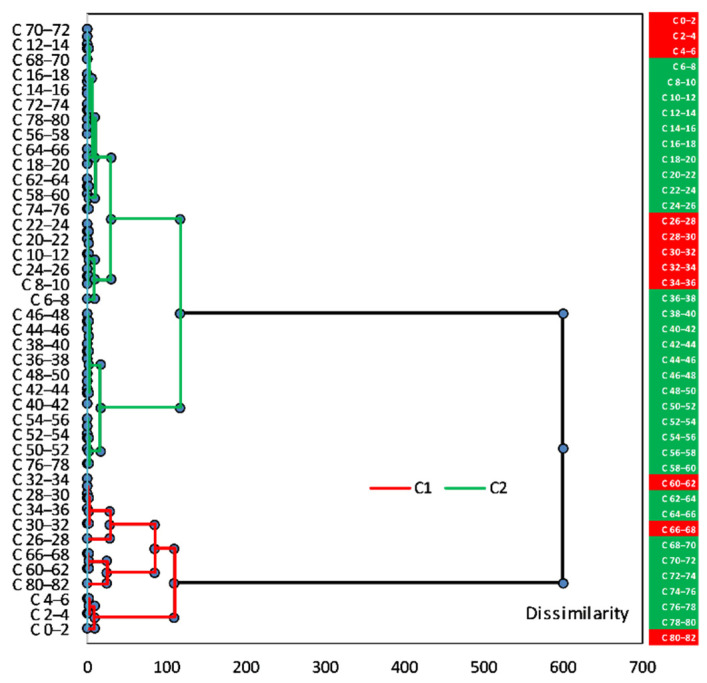
Dendrogram showing the clustering and vertical distribution (on depth) for the sediment sections of Caineni Lake.

Agglomerative hierarchical clustering (AHC) analysis was applied to the principal components score of sediment core sections, i.e., the 41 sections (from C 0–2 cm to C 80–82 cm) in which investigated elements were analysed, to group similar sections (vertical variability). The AHC results were rendered as a dendrogram (Figure 25), where all the sediment sections were clustered in two statistically significant groups/clusters—inertia decomposition for the optimal classification: within-cluster 44.80% and between-clusters 55.20%, H(k − 1)–H(k) at 35.91, the Hartigan index (H) of a clustering with k clusters and a clustering with (k − 1) clusters.

Based on the statistical approach to dataset, the two clusters generated, C1 (within-cluster variance 27.02) and C2 (within-cluster variance 7.47), can provide a representation of the sediment core stratification. An alternation between the layers that group sections belonging to the same cluster, suggesting weathering events that can lead to alternating depositional sequences, can be observed.

**Figure 26:**
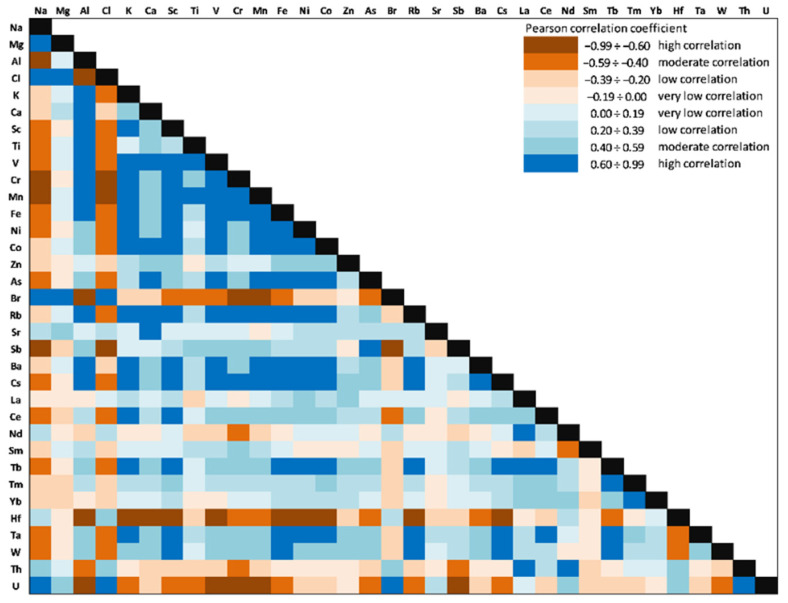
Pearson correlation map based on coefficient matrix for the relationship between the element concentrations in the Movila Miresii Lake sediment core.

Figure 26 highlights the correlations between the 34 investigated elements (Person correlation coefficients) in the Movila Miresii Lake sediment core (90 cm depth).

Significantly positive correlations (significance level alpha = 0.05) for HMs (V: 0.94, 0.70, 0.71, 0.67; Cr: 0.79, 0.66, 0.43, 0.57; Ni: 0.58, 0.74, 0.55; Co: 0.59, 0.69, 0.64) with Al, K, Ca, and Ti, respectively, suggest their primary source is the lithological one. Ba and Cs also show significantly positive correlations (ranged from 0.57÷0.98 and 0.57÷0.86) with Al, K, Ca, Mn, Fe, and HMs.

High values of Pearson coefficients (positive correlation) for LREEs and HREEs were obtained, especially for Ce (0.60, 0.61, and 0.59) and Tb (0.62, 0.80, and 0.65) with K, Sc, and La. As was significantly positively correlated with Ca (0.60), Sc (0.58), V (0.61), Mn (0.63), Fe (0.67), Ni (0.67), and Co (0.76).

**Figure 27:**
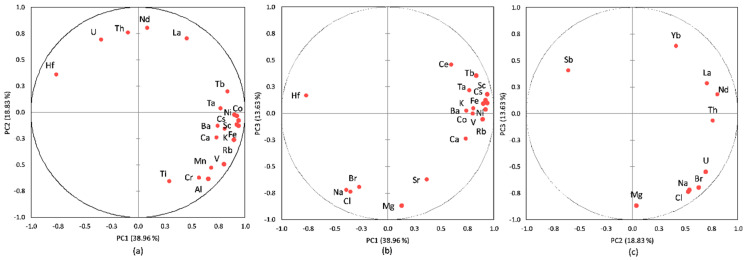
PCA box plots, PC1 versus PC2 (**a**), PC1 versus PC3 (**b**), and PC2 versus PC3 (**c**) obtained for quantified elements in sediment sample of Movila Miresii Lake.

The PCA performed on the database aimed to identify the most important sources for HMs, REEs, and TEs in the sediment core of Movila Miresii Lake. The results from the PCA applied to the database are presented in Figure 27 and Table 8. The first three principal components with eigenvalues greater than 1.00 were selected, with 71.42% of the variance explained. The measure of sampling adequacy by KMO statistics provided the value of 0.69, while the significance of Bartlett’s test of sphericity was less than 0.001, which led to the conclusion that the database size was suitable for evaluation by the PCA. Table 8 summarizes the loading values (classified as strong: loadings values > 0.60, and moderate with loading values 0.60 ÷ 0.30) for the first three principal components.

Figure 27 presents the PCA box plots (correlations between variables and the principal components after Varimax rotation), with the loading of PC1 versus PC2, PC1 versus PC3, and PC2 versus PC3 from the PCA applied to the investigated dataset.

The PC1 versus PC2 box plot (Figure 27a) allows correlating, due to the PC1 strong positive loadings, Al, K, Ca, Mn, Fe, HMs and Sc, Tb, and Ce from REEs. PC1 can be attributed to leaching from the soil surface/denudation, rock weathering, dry or wet atmospheric deposition, and mixed anthropogenic input. PC2 has significant negative loadings for Ti, Cr, Sb, and Sm and high positive loadings for La, Nd, Th, and U. This pattern, together with PC3 moderate/high loading of Tm and Yb, respectively, suggests a mixed contribution from both pedological and anthropogenic inputs. PC3 highlights strong negative loadings for Na, Cl, Br, Sr, and Mg; this principal component can be attributed mainly to salts deposition and denudation.

**Figure 28:**
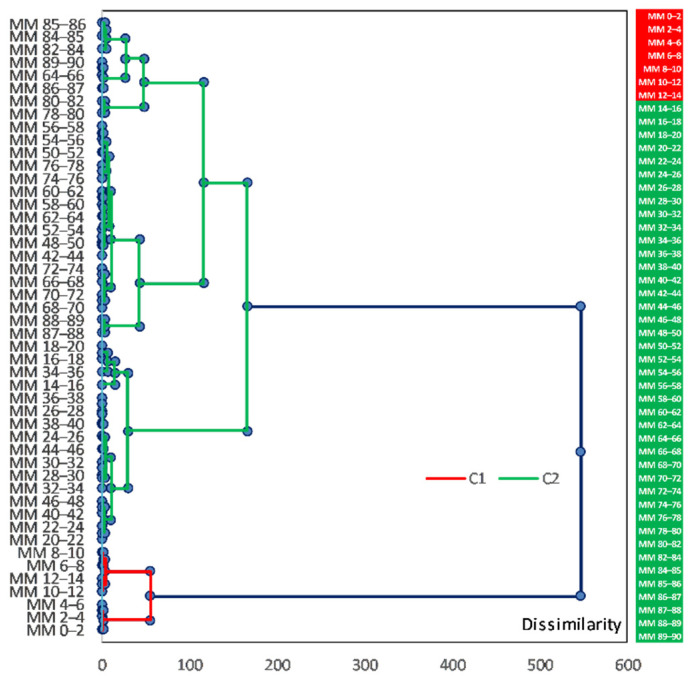
Dendrogram showing the clustering and vertical distribution (on depth) for the sediment sections of Movila Miresii Lake.

PCA analysis was followed by AHC, which was applied to the factors/principal components score of the sediment core sections, i.e., the 41 sections (from MM 0–2 cm to MM 89–90 cm) in which investigated elements were analysed, in order to group similar sections (vertical variability). The AHC results were rendered as a dendrogram (Figure 28), where all the sediment sections were clustered in two statistically significant groups/clusters—inertia decomposition for the optimal classification: within-cluster 52.07% and between-clusters 47.93%, H(k − 1)–H(k) at 24.95, the Hartigan index (H) of a clustering with k clusters and a clustering with (k − 1) clusters.

Based on the statistical approach to the dataset, the two clusters generated, C1 (within-cluster variance 10.90) and C2 (within-cluster variance 13.22), can provide a representation of the sediment core stratification. A clear differentiation between two regions of the sediment core can be observed: upper area included in C1 (from sections MM 0–2 cm to MM 12–14 cm) and bottom area (from sections MM 14–16 cm to MM 89–90 cm). The vertical distribution based on AHC analysis suggests a higher stability in terms of elemental composition and elements migration without significant geochemical or weathering events.

## 4. Conclusions

The variation of HMs, TEs, and REEs in the investigated bottom sediments of the lakes located in the northeast of the Romanian Plain (Lake Amara, Lake Caineni, and Lake Movila Miresii) is mainly due to the specific geological structure of this area, which determines the geochemical processes. The concentration of the investigated elements showed variability dependent on the depth sections and the genesis of the lake and geochemical characteristics (oxbow, fluvial harbor/liman, and loess saucer type). The assessment of pollution indices (contamination factor, pollution load index, geoaccumulation index, and enrichment factor) highlighted low and moderate degrees of contamination for most of the investigated elements. The principal components extracted by PCA statistical analysis were assigned to pedological contribution, salts depositions (due to the lack of a constant supply of freshwater and through evaporation during dry periods), atmospheric deposition, leaching from soil surface/denudation, rock weathering, and mixed anthropogenic input. However, further investigations are still needed, related to saltwater composition, the surrounding geochemical area, and different lithologies, in order to obtain supplementary information. Research will continue in this line.

## Figures and Tables

**Table 1:** Pollution indices and evaluation criteria used for the potential contamination of sediment core.

Pollution Index	Calculation Relationship	Evaluation Criteria	References
Contamination Factor (CF)	CF = C_n_/GBV_n_	<1, low degree of contamination	[29,34,63,64]
		1 ÷ 3, moderate degree of contamination	
		3 ÷ 6, considerable degree of contamination	
		>6, very high degree of contamination	
Pollution Load Index (PLI)	PLI = (CF_1_·CF_2_·…·CF_n_)^1/n^	0, no pollution	[28,30,34]
		0 ÷ 1, only baseline pollutants	
		>1, progressive deterioration	
Geoaccumulation Index (I_geo_)	I_geo_ = log_2_ [C_n_/(1.5·GBV_n_)]	≤0, no contamination	[24,33,34,65]
		0 ÷ 1, none to moderate contamination	
		1 ÷ 2, moderate contamination	
		2 ÷ 3, moderate to heavy contamination	
		3 ÷ 4, heavy contamination	
		4 ÷ 5, heavy to extreme contamination	
		>5, extreme contamination	
Enrichment Factor (EF)	EF = (C_n_/C_Mn_)/(GBV_n_/GBV_Mn)_	0 ÷ 1.5, natural processes	[24,26,27,29,32,34]
		1.5 ÷ 3, minor anthropogenic modification	
		3 ÷ 5, moderate anthropogenic modification	
		5 ÷ 10, severe anthropogenic modification	
		>10, very severe anthropogenic modification	

Notation: C_n_—measured concentration of the element n in the sediment core; GBV_n_—geochemical background concentration of element n; GBV_Mn_—geochemical background concentration of Mn.

**Table 2:** Elements concentration in the Amara Lake bottom sediment core (83–94 cm) in mg·kg^−1^. N normal distribution, LN log-normal distribution.

Element	Min	Mean	Max	SD	Median	Outliers *	Distribution Type	MAD
Main elements								
Na	7940.00	8511.67	10,700.00	1077.88	8070.00	1 ^c^	LN	90.00
Mg	30500.00	31,950.00	33,800.00	1070.98	31,950.00	2 ^c,e^	N	250.00
Al	60,500.00	64,233.33	67,600.00	2647.77	64,000.00		LN	2150.00
Cl	239.00	289.50	427.00	71.32	265.50	1 ^d^	LN	26.00
K	14,800.00	16,350.00	17,700.00	997.50	16,450.00		N	550.00
Ca	42,200.00	45,350.00	51,000.00	3140.54	44,400.00	1 ^d^	LN	1450.00
Ti	4320.00	5123.33	5600.00	435.83	5160.00	1 ^b^	N	140.00
Mn	947.00	1049.50	1140.00	72.12	1050.00		N	45.00
Fe	24,600.00	25,550.00	27,200.00	896.10	25,400.00	1 ^a^	LN	350.00
Br	6.29	6.49	6.64	0.15	6.54		N	0.09
Heavy metals								
V	88.30	95.73	102.00	5.06	97.10		N	3.25
Cr	95.60	103.28	111.00	5.84	102.50		N	4.95
Ni	43.10	44.95	47.60	1.80	44.55		N	1.35
Co	11.00	12.22	13.30	0.75	12.25	2 ^a,e^	N	0.25
Zn	53.80	59.13	63.00	3.33	59.45		N	2.05
As	8.49	9.09	10.30	0.67	8.92	1 ^a^	N	0.35
LREEs and HREEs								
La	33.30	34.62	36.00	1.14	34.80		N	0.90
Ce	60.20	63.37	67.10	2.29	63.35	1 ^a^	N	1.00
Nd	25.60	27.03	28.80	1.30	26.65		LN	0.80
Sm	5.29	5.49	5.60	0.11	5.49	1 ^e^	N	0.06
Tb	0.81	0.85	0.87	0.02	0.85		N	0.02
Tm	0.31	0.36	0.41	0.04	0.37		N	0.03
Yb	2.37	2.69	2.97	0.20	2.70	1 ^d^	N	0.09
Sc	11.30	11.50	11.80	0.18	11.45		N	0.10
Trace elements								
Rb	86.60	88.28	90.30	1.46	88.10		N	1.10
Sr	184.00	197.17	209.00	10.32	201.00		N	5.00
Sb	0.87	0.94	1.18	0.12	0.90	1 ^c^	LN	0.02
Ba	201.00	390.00	510.00	122.72	404.00		N	91.50
Cs	4.38	4.44	4.51	0.05	4.44		N	0.03
Hf	8.56	8.96	9.36	0.37	8.95		N	0.38
Ta	0.96	0.99	1.04	0.04	0.99		N	0.03
W	1.32	1.52	1.60	0.10	1.55	1 ^d^	LN	0.04
Th	9.73	10.04	10.50	0.33	9.90		N	0.14
U	3.02	3.17	3.38	0.13	3.12		N	0.07

* Number of outliers (Grubbs test); a = (83–85), b = (85–87), c = (87–89), d = (91–94), e = (93–94)—the bottom sediment core section [cm].

**Table 3:** Elements concentration in the Caineni Lake bottom sediment core (72–82 cm) in mg·kg^−1^. N normal distribution, LN log-normal distribution.

Element	Min	Mean	Max	SD	Median	Outliers *	Distribution Type	MAD
Main elements								
Na	4970.00	10,514.00	13,100.00	3188.26	11,500.00	1 ^a^	LN	400.00
Mg	44,000.00	45,740.00	48,200.00	1666.73	1100.00		N	1100.00
Al	80,400.00	83,340.00	85,500.00	1907.35	83,400.00		LN	1000.00
Cl	9810.00	10,482.00	11,100.00	523.09	10,400.00		N	500.00
K	10,900.00	23,300.00	29,600.00	7196.87	25,700.00	1 ^a^	LN	1300.00
Ca	39,800.00	43,980.00	51,800.00	4817.36	42,500.00		N	2600.00
Ti	4370.00	4542.00	4750.00	147.21	4510.00		N	110.00
Mn	862.00	918.80	971.00	46.98	911.00		N	49.00
Fe	19,300.00	40,140.00	49,500.00	12,119.94	44,600.00	1 ^a^	LN	4200.00
Br	9.27	19.25	24.20	5.76	21.10	1 ^a^	LN	0.60
Heavy metals								
V	156.00	158.60	162.00	2.41	158.00		N	2.00
Cr	72.70	136.74	170.00	37.35	149.00		LN	8.00
Ni	30.50	70.56	90.80	23.51	80.20		LN	9.60
Co	7.39	16.20	20.40	5.12	18.00	1 ^a^	LN	1.50
Zn	44.10	88.92	110.00	26.61	99.50		LN	10.50
As	6.17	11.49	14.30	3.38	12.20		LN	2.10
LREEs and HREEs								
La	15.90	34.40	43.70	10.73	38.00	1 ^a^	LN	2.10
Ce	27.10	60.82	73.10	19.38	70.70	1 ^a^	LN	2.40
Nd	8.60	25.64	33.80	10.68	30.30		LN	3.50
Sm	2.21	4.72	5.94	1.45	5.13	1 ^a^	LN	0.14
Tb	0.36	0.76	0.91	0.23	0.83	1 ^a^	LN	0.03
Tm	0.21	0.34	0.42	0.09	0.36		LN	0.06
Yb	1.41	2.59	3.67	0.92	2.67		LN	0.69
Sc	8.06	17.49	22.20	5.46	19.30	1 ^a^	LN	0.90
Trace elements								
Rb	70.50	151.30	187.00	46.36	168.00		LN	10.00
Sr	74.30	168.86	212.00	54.08	187.00	1 ^a^	LN	4.00
Sb	0.51	1.12	1.49	0.37	1.21		LN	0.15
Ba	173.00	286.40	402.00	86.88	299.00		LN	63.00
Cs	4.38	9.68	11.90	3.03	10.80	1 ^a^	LN	0.70
Hf	1.17	2.47	3.03	0.74	2.70	1 ^a^	LN	0.11
Ta	0.42	0.94	1.19	0.30	1.03	1 ^a^	LN	0.04
W	0.69	1.61	2.02	0.53	1.81	1 ^a^	LN	0.11
Th	4.65	10.13	12.70	3.16	11.30	1 ^a^	LN	0.70
U	1.19	2.60	3.20	0.80	2.88	1 ^a^	LN	0.08

* Number of outliers (Grubbs test); a = (80–82)—the bottom sediment core section [cm].

**Table 4:** Elements concentration in the Movila Miresii Lake bottom sediment core (80–90 cm) in mg·kg^−1^. N normal distribution, LN log-normal distribution.

Element	Min	Mean	Max	SD	Median	Outliers *	Distribution Type	MAD
Main elements								
Na	11,200.00	11,587.50	12,200.00	335.68	11,500.00		N	200.00
Mg	24,400.00	28,075.00	34,400.00	3193.63	27,800.00	1 ^d^	N	2100.00
Al	48,000.00	59,237.50	70,500.00	7746.32	60,950.00		LN	6100.00
Cl	1750.00	2142.50	2690.00	297.55	2120.00		N	180.00
K	14,600.00	17,025.00	19,800.00	1791.85	16,750.00		N	1050.00
Ca	44,400.00	51,862.50	58,500.00	6183.60	52,250.00		LN	5400.00
Ti	3130.00	3675.00	4440.00	456.04	3575.00		N	365.00
Mn	776.00	1049.63	1360.00	207.59	1008.00		N	138.00
Fe	21,800.00	30,700.00	40,200.00	6257.57	30,100.00		N	4450.00
Br	8.57	9.98	12.00	1.19	9.60		N	0.63
Heavy metals								
V	68.30	89.86	115.00	16.13	89.40		N	13.10
Cr	67.20	82.50	93.30	9.80	81.10		N	9.40
Ni	29.10	41.76	53.30	9.10	42.15		LN	7.30
Co	9.14	13.42	17.80	2.96	13.00		N	2.05
Zn	4.58	61.04	82.80	24.40	66.75	1 ^b^	LN	7.50
As	7.13	13.22	18.20	3.31	12.80		N	1.75
LREEs and HREEs								
La	22.10	25.05	27.40	1.82	24.95		N	1.10
Ce	50.00	57.48	64.60	5.07	57.50		LN	3.40
Nd	13.40	21.34	25.30	3.66	21.25	1 ^a^	N	1.55
Sm	3.53	4.42	5.05	0.45	4.41	1 ^c^	N	0.18
Tb	0.64	0.68	0.74	0.04	0.68		N	0.02
Tm	0.28	0.33	0.38	0.03	0.33		N	0.03
Yb	2.18	2.47	2.70	0.17	2.46		N	0.07
Sc	9.08	10.67	12.60	1.36	10.65		N	1.23
Trace elements								
Rb	60.90	74.79	88.40	8.93	75.20		LN	6.45
Sr	271.00	316.25	355.00	30.04	316.00		N	22.00
Sb	0.79	1.00	1.75	0.31	0.91	1 ^b^	LN	0.05
Ba	312.00	437.50	689.00	143.67	373.50		LN	51.50
Cs	3.57	4.59	5.73	0.79	4.57		N	0.68
Hf	5.45	6.66	8.27	1.14	6.42		N	0.71
Ta	0.74	0.79	0.87	0.05	0.79		N	0.04
W	1.22	1.36	1.51	0.11	1.35		N	0.09
Th	8.10	9.17	10.50	0.87	9.09		N	0.74
U	1.94	2.22	2.42	0.16	2.25		LN	0.08

* Number of outliers (Grubbs test); a = (80–82), b = (84–85), c = (85–86), d = (87–88)—the bottom sediment core section [cm].

**Table 5:** The geochemical background value (GBV) calculated by M2MAD method.

Element	GBV [mg·kg^−1^]		
	Amara	Caineni	Movila Miresii
Na	8250.00	12,300.00	11,900.00
Mg	32,450.00	3300.00	32,000.00
Al	68,300.00	85,400.00	73,150.00
Cl	317.50	11,400.00	2480.00
K	17,550.00	28,300.00	18,850.00
Ca	47,300.00	47,700.00	63,050.00
Ti	5440.00	4730.00	4305.00
Mn	1140.00	1009.00	1284.00
Fe	26,100.00	53,000.00	39,000.00
Br	6.71	22.30	10.86
V	103.60	162.00	115.60
Cr	112.40	165.00	99.90
Ni	47.25	99.40	56.75
Co	12.75	21.00	17.10
Zn	63.55	120.50	81.75
As	9.62	16.40	16.30
La	36.60	42.20	27.15
Ce	65.35	75.50	64.30
Nd	28.25	37.30	24.35
Sm	5.61	5.41	4.76
Tb	0.89	0.89	0.72
Tm	0.44	0.48	0.38
Yb	2.87	4.05	2.60
Sc	11.65	21.10	13.10
Rb	90.30	188.00	88.10
Sr	211.00	195.00	360.00
Sb	0.93	1.51	1.01
Ba	587.00	425.00	476.50
Cs	4.51	12.20	5.92
Hf	9.71	2.92	7.84
Ta	1.06	1.11	0.87
W	1.63	2.03	1.53
Th	10.18	12.70	10.57
U	3.25	3.04	2.41

**Table 6:** Principal component analysis data for the elements quantified in sediment sample of Amara Lake.

Element	KMO	PC1		PC2		PC3	
		Loadings	Squared Cosine	Loadings	Squared Cosine	Loadings	Squared Cosine
Na	0.74	0.17	0.03	0.72	**0.51**	−0.25	0.06
Mg	0.80	−0.11	0.01	−0.81	**0.66**	0.37	0.13
Al	0.67	−0.21	0.04	−0.73	**0.54**	0.44	0.19
Cl	0.70	0.10	0.01	0.91	**0.82**	−0.25	0.06
K	0.71	0.07	0.00	−0.07	0.01	0.85	**0.72**
Ca	0.82	−0.73	**0.54**	−0.08	0.01	0.35	0.12
Sc	0.80	−0.23	0.05	−0.29	0.08	0.87	**0.75**
Ti	0.70	0.01	0.00	−0.69	**0.47**	0.02	0.00
V	0.73	−0.30	0.09	−0.71	**0.50**	0.53	0.28
Cr	0.71	0.19	0.04	0.72	**0.51**	0.35	0.12
Mn	0.53	0.20	0.04	−0.49	**0.24**	0.40	0.16
Fe	0.65	−0.07	0.00	0.22	0.05	0.88	**0.77**
Ni	0.74	−0.38	0.14	−0.19	0.03	0.85	**0.73**
Co	0.89	−0.19	0.04	−0.28	0.08	0.87	**0.76**
Zn	0.57	−0.52	0.27	0.58	**0.33**	0.42	0.18
As	0.47	0.68	**0.47**	−0.04	0.00	0.11	0.01
Br	0.79	0.02	0.00	0.94	**0.89**	−0.05	0.00
Rb	0.72	−0.17	0.03	−0.26	0.07	0.88	**0.78**
Sr	0.79	−0.06	0.00	0.93	**0.86**	−0.03	0.00
Sb	0.49	−0.02	0.00	0.07	0.00	0.69	**0.47**
Ba	0.71	−0.40	**0.16**	0.11	0.01	0.17	0.03
Cs	0.72	−0.35	0.12	−0.30	0.09	0.84	**0.71**
La	0.81	0.94	**0.87**	0.00	0.00	−0.13	0.02
Ce	0.80	0.90	**0.82**	0.19	0.04	−0.18	0.03
Nd	0.72	0.64	**0.40**	0.01	0.00	−0.04	0.00
Sm	0.61	0.77	**0.60**	0.18	0.03	0.08	0.01
Tb	0.73	0.84	**0.70**	−0.04	0.00	−0.01	0.00
Tm	0.63	0.34	**0.12**	0.06	0.00	−0.11	0.01
Yb	0.79	0.71	**0.50**	0.24	0.06	−0.41	0.16
Hf	0.76	0.63	**0.40**	−0.12	0.01	−0.58	0.34
Ta	0.60	0.74	**0.55**	0.14	0.02	−0.04	0.00
W	0.59	0.07	0.01	0.87	**0.76**	0.13	0.02
Th	0.83	0.94	**0.88**	0.13	0.02	−0.17	0.03
U	0.66	0.45	**0.20**	0.42	0.18	−0.44	0.19
Eigenvalue		12.72		6.53		4.66	
Variability [%]		37.42		19.19		13.72	
Cumulative [%]		37.42		56.61		70.33	
Variability [%] *		24.01		23.21		23.11	
Cumulative [%] *		24.01		47.22		70.33	

Note: KMO: Kaiser–Meyer–Olkin measure of sampling adequacy; Loadings and squared cosines after Varimax rotation (values in bold correspond for each variable to the principal component for which the squared cosine is the largest); * Varimax rotation (Kaiser normalization).

**Table 7:** Principal component analysis data for the elements quantified in sediment sample of Caineni Lake.

Element	KMO	PC1		PC2		PC3	
		Loadings	Squared Cosine	Loadings	Squared Cosine	Loadings	Squared Cosine
Na	0.47	0.50	0.25	0.72	**0.51**	−0.30	0.09
Mg	0.85	0.35	0.13	−0.09	0.01	0.84	**0.71**
Al	0.71	0.41	0.17	−0.19	0.04	0.85	**0.73**
Cl	0.60	−0.21	0.04	0.83	**0.68**	0.19	0.04
K	0.86	0.84	**0.71**	0.11	0.01	0.31	0.10
Ca	0.79	−0.43	0.19	0.73	**0.53**	−0.21	0.05
Sc	0.86	0.88	**0.78**	−0.16	0.03	0.34	0.11
Ti	0.81	0.30	0.09	−0.33	0.11	0.77	**0.59**
V	0.79	0.42	0.17	−0.09	0.01	0.88	**0.77**
Cr	0.93	0.84	**0.71**	−0.21	0.04	0.31	0.10
Mn	0.44	−0.13	0.02	0.76	**0.58**	0.24	0.06
Fe	0.83	0.89	**0.80**	−0.01	0.00	0.27	0.07
Ni	0.86	0.90	**0.82**	−0.22	0.05	0.18	0.03
Co	0.84	0.86	**0.74**	−0.22	0.05	−0.01	0.00
Zn	0.76	0.86	**0.75**	−0.11	0.01	0.05	0.00
As	0.70	0.61	**0.38**	0.00	0.00	0.19	0.04
Br	0.62	−0.19	0.03	0.87	**0.75**	−0.42	0.17
Rb	0.78	0.88	**0.77**	−0.14	0.02	0.36	0.13
Sr	0.72	−0.40	0.16	0.77	**0.60**	−0.38	0.14
Sb	0.81	0.82	**0.67**	−0.30	0.09	0.14	0.02
Ba	0.65	0.63	**0.40**	−0.09	0.01	−0.06	0.00
Cs	0.79	0.84	**0.71**	−0.10	0.01	0.43	0.19
La	0.73	0.87	**0.75**	0.00	0.00	0.33	0.11
Ce	0.90	0.94	**0.89**	−0.12	0.01	0.11	0.01
Nd	0.76	0.71	**0.50**	−0.30	0.09	0.05	0.00
Sm	0.86	0.91	**0.82**	0.05	0.00	0.18	0.03
Tb	0.86	0.91	**0.82**	−0.25	0.06	0.12	0.01
Tm	0.76	0.64	**0.40**	−0.49	0.24	0.01	0.00
Yb	0.65	0.57	**0.33**	−0.42	0.18	−0.27	0.07
Hf	0.35	0.23	0.05	−0.36	0.13	−0.67	**0.45**
Ta	0.80	0.89	**0.80**	−0.23	0.05	0.18	0.03
W	0.78	0.82	**0.68**	−0.18	0.03	0.12	0.01
Th	0.84	0.94	**0.89**	−0.09	0.01	0.14	0.02
U	0.59	0.08	0.01	0.94	**0.89**	−0.12	0.02
Eigenvalue		19.00		4.69		3.48	
Variability [%]		55.88		13.80		10.24	
Cumulative [%]		55.88		69.68		79.92	
Variability [%] *		48.27		17.20		14.45	
Cumulative [%] *		48.27		65.47		79.92	

Note: KMO: Kaiser–Meyer–Olkin measure of sampling adequacy; Loadings and squared cosines after Varimax rotation (values in bold correspond for each variable to the principal component for which the squared cosine is the largest); * Varimax rotation (Kaiser normalization).

**Table 8:** Principal component analysis data for the elements quantified in sediment sample of Movila Miresii Lake.

Element	KMO	PC1		PC2		PC3	
		Loadings	Squared Cosine	Loadings	Squared Cosine	Loadings	Squared Cosine
Na	0.60	−0.35	0.12	0.53	0.28	−0.74	**0.54**
Mg	0.31	0.13	0.02	0.04	0.00	−0.87	**0.75**
Al	0.72	0.66	**0.43**	−0.63	0.40	0.10	0.01
Cl	0.65	−0.39	0.15	0.54	0.29	−0.72	**0.52**
K	0.86	0.81	**0.65**	−0.15	0.02	0.05	0.00
Ca	0.68	0.73	**0.54**	−0.24	0.06	−0.24	0.06
Sc	0.80	0.94	**0.88**	−0.13	0.02	0.18	0.03
Ti	0.77	0.29	0.08	−0.66	**0.43**	0.05	0.00
V	0.79	0.80	**0.64**	−0.50	0.25	0.00	0.00
Cr	0.71	0.57	0.32	−0.62	**0.39**	0.21	0.05
Mn	0.70	0.68	**0.47**	−0.53	0.28	0.16	0.03
Fe	0.81	0.92	**0.85**	−0.12	0.01	0.12	0.02
Ni	0.90	0.90	**0.81**	−0.02	0.00	0.09	0.01
Co	0.82	0.92	**0.85**	−0.03	0.00	0.03	0.00
Zn	0.57	0.51	**0.26**	0.20	0.04	0.09	0.01
As	0.63	0.63	**0.39**	−0.30	0.09	0.23	0.05
Br	0.75	−0.27	0.07	0.63	0.39	−0.69	**0.48**
Rb	0.80	0.89	**0.80**	−0.26	0.07	−0.06	0.00
Sr	0.36	0.37	0.13	0.07	0.01	−0.62	0.39
Sb	0.65	0.21	0.04	−0.61	**0.37**	0.41	0.17
Ba	0.82	0.74	**0.55**	−0.13	0.02	0.03	0.00
Cs	0.74	0.94	**0.88**	−0.08	0.01	0.10	0.01
La	0.52	0.46	0.21	0.71	**0.50**	0.29	0.08
Ce	0.64	0.60	**0.36**	0.16	0.03	0.46	0.21
Nd	0.61	0.08	0.01	0.80	**0.64**	0.18	0.03
Sm	0.39	−0.05	0.00	−0.60	**0.36**	0.02	0.00
Tb	0.74	0.84	**0.70**	0.20	0.04	0.35	0.13
Tm	0.56	0.44	0.19	0.19	0.04	0.47	**0.22**
Yb	0.51	0.26	0.07	0.41	0.17	0.64	**0.41**
Hf	0.62	−0.77	**0.59**	0.36	0.13	0.17	0.03
Ta	0.69	0.77	**0.59**	0.04	0.00	0.22	0.05
W	0.61	0.67	**0.45**	−0.11	0.01	0.22	0.05
Th	0.55	−0.09	0.01	0.76	**0.58**	−0.07	0.00
U	0.61	−0.34	0.12	0.70	**0.48**	−0.55	0.30
Eigenvalue		15.40		5.20		3.67	
Variability [%]		45.31		15.31		10.80	
Cumulative [%]		45.31		60.62		71.42	
Variability [%] *		38.96		18.83		13.63	
Cumulative [%] *		38.96		57.79		71.42	

Note: KMO: Kaiser–Meyer–Olkin measure of sampling adequacy; Loadings and squared cosines after Varimax rotation (values in bold correspond for each variable to the principal component for which the squared cosine is the largest); * Varimax rotation (Kaiser Normalization).

## Data Availability

The data reported in this study are available on request from the first author.
